# Nonlinear Measures Applied to Spontaneous Infant Movement Analysis: A Scoping Review

**DOI:** 10.3390/s26134267

**Published:** 2026-07-04

**Authors:** Joana Ferreira, Marta Freitas, Sofia Gaspar, Francisco Pinho, Hélder Fonseca, Cláudia Silva

**Affiliations:** 1 Research Center in Physical Activity, Health and Leisure (CIAFEL), Faculty of Sport, University of Porto (FADEUP), 4200-450 Porto, Portugal; hfonseca@fade.up.pt; 2 Laboratory for Integrative and Translational Research in Population Health (ITR), 4200-450 Porto, Portugal; 3 CIR, ESS, Polytechnic of Porto, Rua Dr. António Bernardino de Almeida No. 400, 4200-072 Porto, Portugal; ccs@ess.ipp.pt; 4 ESS, Polytechnic of Porto, Rua Dr. António Bernardino de Almeida No. 400, 4200-072 Porto, Portugal; 10230092@ess.ipp.pt; 5 Escola Superior de Saúde do Vale do Ave, Cooperativa de Ensino Superior Politécnico e Universitário, 4760-409 Vila Nova de Famalicão, Portugal; marta.goncalves@ipsn.cespu.pt (M.F.); francisco.pinho@ipsn.cespu.pt (F.P.); 6 H2M-Health and Human Movement Unit, Instituto Politécnico de Saúde do Norte, CESPU, CRL, 4760-409 Vila Nova de Famalicão, Portugal

**Keywords:** early motor development, neurodevelopmental assessment, movement variability, motor complexity

## Abstract

Spontaneous movement analysis provides valuable information about the maturation of the central nervous system and the emergence of motor control strategies in very young babies. Nonlinear measures capture dynamic aspects of movement that cannot be represented by linear methods. However, their implementation in clinical practice faces challenges, including the lack of standardized protocols and accessible tools for routine use. This scoping review aimed to map and characterize the nonlinear measures used to analyze spontaneous infant movement, including assessment context, instruments, data collection protocols, and main variables. The review followed JBI methodology and PRISMA-ScR guidelines. Searches were conducted in PubMed^®^, Web of Science™, IEEE Xplore^®^, ScienceDirect^®^, and Google Scholar for studies published from 1 January 2005 to 31 December 2025. Of 1166 records identified, 18 met the inclusion criteria. The nonlinear measures were grouped into five main methodological families: entropy-based measures (*n* = 10), state-space and dynamical systems measures (*n* = 4), recurrence-based analysis (*n* = 3), symbolic and discrete-state approaches (*n* = 3), and variance and frequency-based nonlinear descriptors (*n* = 1). Studies were conducted in laboratory settings (*n* = 6) and in hospital and/or home environments (*n* = 10). Two studies did not clearly specify the assessment context. Kinematic assessment was mainly performed using video-based systems (*n* = 7), accelerometers (*n* = 4), and wearable sensors (*n* = 2), with most studies focusing on the upper and lower limbs. Several investigations extended beyond single-joint analyses to examine inter-limb relationships and whole-body configurations, capturing spatial coordination patterns across multiple body segments. Kinetic assessment was conducted using pressure mats (*n* = 4) and force platforms (*n* = 1), with the center of pressure displacement as the primary outcome. Future research should prioritise methodological harmonisation and theoretical clarity. Consensus is needed regarding minimal data requirements, parameter selection, and reporting standards for commonly used nonlinear measures. Studies should also move beyond single-metric approaches and adopt multivariate frameworks that integrate complementary nonlinear metrics. The absence of standardised acquisition and analytical protocols currently limits cross-study comparability and hinders the clinical translation of nonlinear movement metrics as objective tools for early neurodevelopmental assessment.

## 1. Introduction

Spontaneous movements (SM) represent a critical window in neuromotor development, providing valuable insights into the maturation of the central nervous system (CNS) and the emergence of motor control strategies [1,2,3]. These intrinsically generated movements typically emerge around the 7th gestational week and gradually evolve into more structured and intentional motor behaviours as the nervous system develops [4]. This progression reflects the activity of subcortical and spinal circuits, along with the maturation of corticospinal pathways, and is considered an early marker of CNS functional integrity [1,2,3,4,5,6,7,8].

General movements (GM), a subtype of SM, emerge around the 9th postmenstrual week and persist until approximately 20–25 weeks post-term [9]. They involve coor-dinated activity of all body segments-limbs, head, and trunk-and are characterized by smooth initiation and termination, as well as variations in amplitude, direction, and velocity [4,9,10,11,12]. Up to 38–40 gestational weeks, GMs are classified as fetal or preterm. From this point until 6–9 weeks post-term, writhing movements (WM) predominate, involving the entire body with small to moderate amplitude, slow to moderate velocity, and gradual onset and offset [6,9,13,14]. Between 11 and 16 weeks post-term, fidgety movements (FM) emerge, characterized by small, continuous, circular movements of low amplitude, persisting until 20–25 weeks [4,10].

GMs are widely recognized for their predictive value in identifying neuromotor abnormalities and are extensively used in infant developmental assessments [1,2,3,5,7,8,11,14,15,16,17,18,19]. The quality and variability of GMs are directly associated with CNS integrity [12,20,21,22]. Complex and variable movement patterns suggest healthy neuro-logical maturation, whereas monotonous, rigid, or low-complexity patterns may indicate motor and neurological developmental impairments [4,12,14,23]. Early identification of atypical motor patterns enables more targeted and potentially more effective therapeutic interventions, optimizing developmental and functional outcomes [5,8,15,17,21,24,25,26]. From a clinical perspective, objective methods capable of quantifying movement organisation may complement observational assessments by reducing dependence on evaluator expertise and enabling the detection of subtle alterations in movement dynamics that may precede overt clinical manifestations of neurodevelop-mental impairment [5,9,15,26]. Quantitative approaches capable of characterising the temporal organisation, complexity, and adaptability of spontaneous movements may provide additional biomarkers of early central nervous system function and contribute to more objective monitoring of developmental trajectories and intervention outcomes [27].

These considerations are particularly relevant because the observational assessment of GMs, despite its well-established clinical value, relies on highly specialised evaluators and is subject to interobserver variability, which may limit its widespread implementation and has motivated the development of more objective and automated assessment approaches [5,9,14,26,27]. Linear approaches, such as means and standard deviations applied to movement amplitude, velocity, frequency, or force, provide objective and informative descriptions of global motor dynamics by quantifying signal magnitude and frequency characteristics that remain clinically relevant [2,7,23,27,28,29,30,31,32,33,34,35,36,37,38,39]. However, these approaches are limited in their ability to characterise the temporal organisation and inherent variability of human movement, which is fundamentally nonlinear in nature. Nonlinear measures provide a complementary framework for investigating movement complexity, regularity, adaptability, and self-organisation over time. Rather than replacing linear analyses, nonlinear metrics extend them by revealing subtle alterations in temporal structure that may remain undetected when only global summary statistics are considered [40,41]. Importantly, these properties may have relevance for infant assessment, as changes in movement complexity and temporal organisation may emerge before gross alterations in movement quantity or amplitude become clinically apparent [41]. Consequently, nonlinear metrics may offer greater sensitivity for identifying early disturbances in motor organisation and for objectively quantifying subtle developmental changes that are difficult to capture through observational assessment alone [40,41,42,43,44].

Metrics such as approximate entropy, fractal dimension, and Lyapunov exponent capture dynamic aspects of movement that linear methods cannot represent [37,45,46,47,48,49,50]. These metrics have been successfully applied in other clinical contexts, such as chronic stroke [51], highlighting their potential to capture the complexity of human movement. Nevertheless, their application to SM analysis in infants remains limited and fragmented [52,53]. Despite growing interest, clinical adoption of these metrics faces practical challenges, including the lack of standardized protocols and accessible tools for integration into routine clinical practice.

Given this context, a scoping review was conducted to map a nascent and heterogeneous body of evidence, synthesize current trends, and identify research priorities. This type of review is particularly relevant in emerging and fragmented fields, such as the application of nonlinear metrics to SM.

The main objective of this scoping review was to map and summarize nonlinear measures used to assess SM in infants up to 6 months of age. Additionally, it aimed to summarize the methodological considerations, namely participant characteristics, assessment context, instruments, sample frequency, data collection protocol and main variables applied. By mapping these approaches, this review seeks to advance the understanding of early motor complexity, support the development of standardized assessment frameworks, and facilitate the clinical integration of nonlinear metrics to enhance early intervention strategies and improve developmental outcomes. Furthermore, this review seeks to critically explore the extent to which the currently available evidence supports the potential clinical applicability of nonlinear measures as objective tools for the assessment of early motor organisation and neurodevelopment.

Review questions

The main review question was “What nonlinear measures have been used in the assessment and characterization of infants’ spontaneous movements?”

The review sub-questions are listed as follows:

1. What were the protocols under which spontaneous movements were recorded, including the assessment context and recording duration, in the included studies?
2. What instruments and acquisition systems were used to collect movement data in the included studies?
3. Which movement-related variables (e.g., kinematic or kinetic) were analyzed in relation to nonlinear measures in the included studies?
4. To what extent does the currently available evidence support the potential clinical applicability of nonlinear measures as objective tools for the assessment of early motor organisation and neurodevelopment in infants?

## 2. Materials and Methods

This scoping review was conducted in accordance with the Preferred Reporting Items for Systematic Reviews and Meta-Analyses—extension for Scoping Reviews (PRISMA-ScR) [54] and followed the methodological recommendations of the Joanna Briggs Institute (JBI) Manual for Evidence Synthesis [55].

The protocol for the Appendix A was registered on the Open Science Framework (OSF), detailing the review questions and methodological procedures (DOI: https://doi.org/10.17605/OSF.IO/UJT5D; available at https://osf.io/ujt5d, accessed on 5 November 2025). Additional supporting information can be retrieved from the same source. Minor refinements introduced during the review process did not affect the review objectives, eligibility criteria, or overall methodological framework.

### 2.1. Eligibility Criteria

The research strategy was defined based on the PCC (Population, Concept, and Context) framework, in accordance with JBI methodology [52] (Table 1).

Articles with observational or experimental study designs published in English, Portuguese, or French between 2005 and 2025 were included, as the systematic empirical application of nonlinear measures to spontaneous infant movement analysis emerged predominantly during this period. The upper boundary (2025) corresponds to the date on which the searches were completed. Systematic reviews, meta-analyses, narrative and scoping reviews (to avoid duplication of data), letters, editorials, qualitative methodological designs and academic dissertations were excluded. Studies that used electroencephalography (EEG) as the primary signal source rather than movement-based analysis were also excluded.

### 2.2. Search Strategy

Searches were conducted for studies published from 1 January 2005 to 31 December 2025. Relevant studies were identified by searching the PubMed^®^, Web of Science™, Institute of Electrical and Electronics Engineers (IEEE Xplore^®^), and ScienceDirect^®^ databases, as well as the Google Scholar search engine, with a maximum of 200 results per query ordered by relevance. Given the inherent reproducibility limitations of this engine, Google Scholar was used as a supplementary strategy to capture records potentially missed by the structured database searches, rather than as a primary retrieval source. To complement the electronic search, a manual analysis of the reference lists of original articles and relevant reviews was also performed, applying the snowballing technique, with the aim of identifying additional potentially eligible studies.

The keywords were combined using Boolean operators, with combinations adjusted to each database. The following search string was used for PubMed^®^ and subsequently adapted to other databases: (infant* OR newborn OR baby* OR neonate) AND (movement OR “spontaneous movement”) AND (nonlinear* OR “non-linear” OR entropy OR Lyapunov OR “complexity” OR “time series”) NOT (EEG OR heart). The complete search strategies for all databases, including the full search strings and applied limits, are provided in Appendix A.1.

Searches were performed independently by two reviewers to ensure reproducibility and transparency.

### 2.3. Selection of Evidence Sources

The selection of evidence sources considered the PCC acronym, purpose, and research questions. The screening process took place in three stages: reading titles, analysing abstracts, and fully evaluating potentially eligible texts. All results identified in the search were imported into Rayyan^®^ software (Rayyan Systems Inc., Cambridge, MA, USA), which allowed for the organization, detection, and removal of duplicates, and the classification of studies as “included” or “excluded” according to the previously defined criteria.

**Figure 1:**
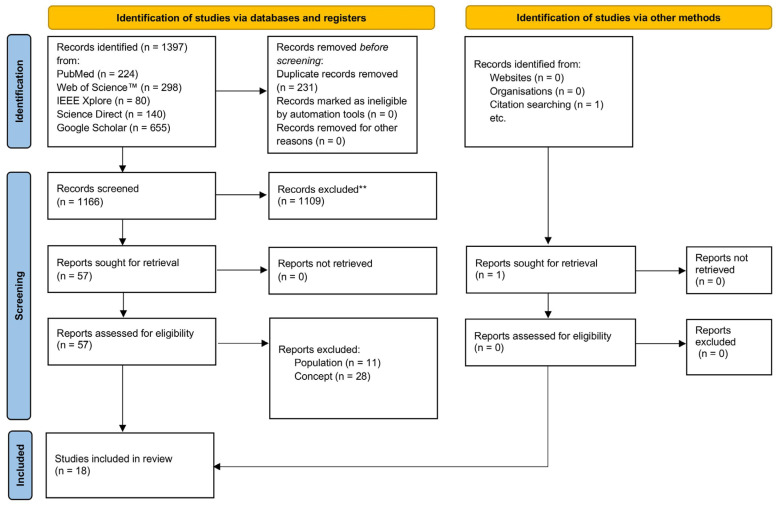
Flowchart of included studies for the scoping review process, adapted from the PRISMA-ScR statement [42].

Before formal screening, a pilot calibration was conducted: both reviewers independently screened the first 50 titles and abstracts to verify consistent interpretation of eligibility criteria. Once an inter-reviewer agreement of ≥75% was achieved, the main screening process began [51]. Any disagreements were resolved by consensus or with the intervention of a third reviewer. A formal inter-reviewer agreement statistic (e.g., Cohen’s kappa) was not calculated following the calibration exercise. The overall selection process is presented in the PRISMA-ScR flow diagram (Figure 1).

### 2.4. Data Extraction

After initial screening, the selected articles were imported into Mendeley^®^—Reference Management Software, version 2.134.0 (Elsevier B.V., Amsterdam, The Netherlands), which supported full-text reading and systematic data extraction. Two reviewers independently extracted data using a charting table adapted from the JBI template, and discrepancies were resolved by consensus or by a third author. The data extraction form was developed according to the review objectives and variables of interest but was not formally piloted prior to its final application.

Data was organized and managed using Microsoft Excel (Microsoft 365). The following categories were collected: general study information (authors, year of publication, country, study design); population characteristics (sample size, sex, gestational and assessment age, birth weight, risk category (e.g., preterm, high-risk or typical development); assessment context (e.g., home, clinical, laboratory); instruments (type of measurement system, e.g., video-based analysis, motion capture, inertial sensors, electromyography); sampling frequency; data collection protocol; nonlinear measures (e.g., sample entropy, approximate entropy, Lyapunov exponent, recurrence quantification analysis) and main variables (e.g., kinematic, kinetic or EMG parameters analysed). Additionally, where reported, information on complementary analytical parameters and computational settings—such as the use of machine learning or dimensionality reduction approaches—was extracted. However, specific technical details including neural network architectures and signal filtering cutoff frequencies were inconsistently reported across the included studies, precluding systematic extraction of these parameters (Appendix A.2).

### 2.5. Data Presentation

A narrative synthesis approach was adopted to collate and present the extracted data according to predefined analytical domains, including the type of nonlinear measure applied, characteristics of the infant population, study context and instruments used for data acquisition, movement-derived signals and variables analysed, and key methodological considerations relevant to the application of nonlinear analysis to spontaneous infant movements.

Descriptive tables were developed to summarize study characteristics and methodological features. These tables complement narrative synthesis and provide an integrated overview of how nonlinear methods have been applied to the analysis of spontaneous infant movements.

No quantitative synthesis or meta-analysis was performed, in accordance with JBI guidance for scoping reviews. The emphasis was placed on mapping the breadth, methodological diversity, and emerging trends within this field.

## 3. Results

A total of 1398 records were identified, including 1397 records retrieved through electronic database and search engine searches and one additional record identified through citation searching. After removal of 231 duplicate records, 1166 records remained for title and abstract screening. Of these, 1109 records were excluded for not meeting the eligibility criteria defined according to the PCC framework.

Fifty-seven reports were sought for full-text retrieval, all of which were successfully retrieved. Following full-text assessment, 39 reports were excluded for not meeting the inclusion criteria, primarily related to population (*n* = 11) and concept (*n* = 28). A total of 18 studies were therefore included in this scoping review. The study selection process is illustrated in the PRISMA-ScR flow diagram (Figure 1).

Table 2 summarizes the main characteristics of the included studies, providing information on participant characteristics, assessment context, instruments, sample frequency, data acquisition protocols, nonlinear measures, and the main variables analysed.

### 3.1. General Characteristics of Included Studies

The included studies were published between 2006 and 2024 and were conducted across diverse geographical regions, including Europe, Asia and North America.

Overall, the literature was dominated by exploratory and observational designs. These comprised cross-sectional studies [23,43,44,56,57,58,66], longitudinal observational studies [59,61,62,63,64], and comparative observational studies involving clinical or at-risk infant groups alongside control groups [45,57,58,61,69].

In addition, two studies adopted an observational methodological design with predictive aims, integrating nonlinear movement metrics with clinical outcome measures [67,69].

**Figure 2:**
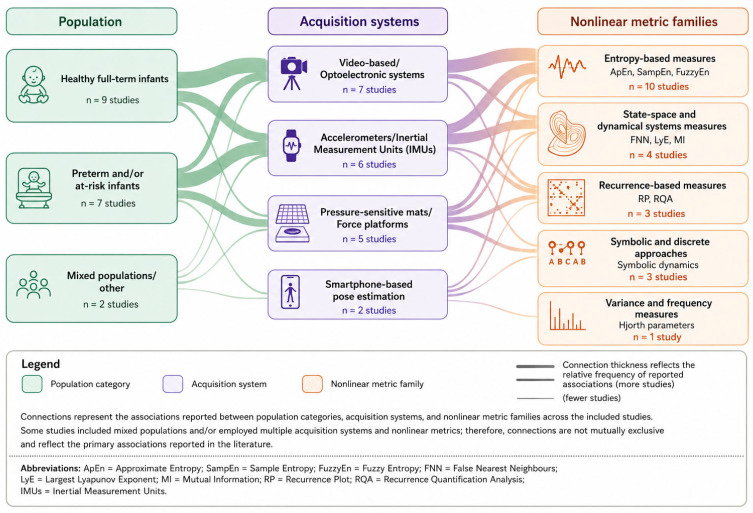
Integrated overview of the relationships between study populations, acquisition systems, and nonlinear metric families across the included studies.

To facilitate the interpretation of the considerable methodological heterogeneity identified across studies, Figure 2 provides an integrated overview of the relationships between study populations, acquisition systems, and nonlinear metric families used to analyse spontaneous infant movements.

### 3.2. Participants

Sample sizes across the included studies ranged from very small cohorts of six infants [54,55,56] to larger samples of up to 90 infants [69]. Studies with larger sample sizes tended to focus either on specific body segments, such as the lower limbs [60,68], or to employ scalable data acquisition approaches, notably smartphone RGB video-based systems combined with automated pose estimation [67,69].

Some studies included exclusively healthy full-term infants (*n* = 8) [43,44,56,59,62,65,66,68] or babies with typical motor development (*n* = 1) [23]. It should be noted that Aßmann et al. [43,44] correspond to two publications derived from the same cohort of healthy full-term newborns, addressing complementary analytical questions rather than independent samples.

Other studies focused on preterm infant populations, frequently characterised by gestational ages below 32 weeks or birth weights under 1500 g [64,67,69], or have compared full-term and preterm babies [58,63]. Several investigations included infants with specific clinical conditions, such as preterm infants with brain injury [57,61] and infants with myelomeningocele (MMC) [60]. Additionally, one study examined infants classified as being at increased risk for developmental delay based on perinatal and developmental criteria [45].

Sex distribution was generally balanced in studies that reported this information [43,45,56,57,58,59,61,62,63,64,65,67,68,69]. However, sex-specific data were not consistently reported across all studies [23,60,66].

The age at assessment varied widely, ranging from the first days of life to approximately six months of corrected age. Several studies focused predominantly on the period of writhing movements [43,44,56,57,58,67,69], whereas others specifically targeted the fidgety movement period [23,64,66]. Longitudinal studies frequently covered developmental windows spanning both writhing and fidgety movements, allowing examination of transitions between these phases [59,60,61,62,63,65,68]. In contrast, one study analysed spontaneous movements across a broad age range without differentiating developmental phases and additionally included voluntary movement behaviours [45]. 

### 3.3. Context

Regarding the assessment context, most studies were conducted in hospital and/or home settings (n = 10) [45,56,57,58,59,61,62,67,68,69]. These contexts were generally selected to reflect infants’ natural environments or routine clinical follow-up conditions, particularly in studies involving preterm infants or those at increased neurological risk.

Six studies assessed spontaneous movements in laboratory settings [23,43,44,60,65,66], typically under highly controlled conditions that allowed precise kinematic or kinetic data acquisition. Within this group, Aßmann et al. [43,44] correspond to two publications derived from the same experimental protocol and cohort, addressing complementary analytical objectives rather than independent samples. In contrast, two studies did not explicitly report the assessment context [63,64], although a home-based setting may be inferred from the protocol characteristics and their methodological similarity to previous studies conducted by the same research group.

Studies with larger sample sizes were predominantly conducted in hospital and/or home settings [45,58,62,67,68,69], reflecting the feasibility and scalability of data collection in less constrained environments. An exception was the study by Smith et al. [60], which, despite a relatively large number of observations, was performed in a laboratory setting.

Overall, Table 2 indicates that studies involving healthy full-term infants were more frequently conducted in laboratory settings [23,43,44,60,65,66], whereas studies including preterm infants or infants at neurological or developmental risk were predominantly performed in hospital or combined hospital–home settings [57,61,67,69]. In several cases, assessments were initiated in hospital settings and subsequently continued at home following clinical stabilisation or discharge, enabling longitudinal monitoring of spontaneous movement development across different developmental stages and environments [61,67,69]. It should be noted that, in two studies [63,64], the assessment context was not explicitly reported and was classified based on protocol characteristics and methodological similarities with previous studies from the same research group.

### 3.4. Instruments and Variables

A wide range of movement acquisition systems was employed across the included studies to capture spontaneous infant movements. Early and exploratory investigations predominantly relied on video-based motion capture approaches, using synchronized multi-camera setups to reconstruct three-dimensional kinematics from two-dimensional recordings [23,43,44,60,65]. These systems enabled detailed characterization of joint kinematics, limb trajectories, and spatial relationships between body segments under controlled laboratory conditions.

Wearable inertial sensors, particularly triaxial accelerometers and inertial measurement units (IMUs), were frequently used to record spontaneous limb movements in hospital and home environments [45,56,57,59,61,68]. These sensors provided continuous time series of linear acceleration and angular velocity, allowing long-duration recordings of spontaneous activity in naturalistic settings.

Some studies employed pressure-sensitive mats [58,62,63,64] or force platforms [66] to quantify CoP displacement and its temporal derivatives during spontaneous movements. These approaches primarily generated kinetic signals reflecting postural sway and weight-shifting behaviour.

Across studies, kinematic analyses predominantly focused on the upper and lower limbs, including shoulder, elbow, wrist, hip, knee, and ankle joints, as well as hand and foot trajectories [23,43,44,60,67,69]. Several investigations extended beyond single-joint analyses to examine inter-limb relationships and whole-body configurations, capturing spatial coordination patterns across multiple body segments [23,43,44,45,65].

More recent investigations increasingly adopted video-based pose estimation techniques, combining conventional or smartphone RGB video recordings with deep learning-based models to extract joint coordinates and kinematic variables [67,69].

Kinetic analyses were limited to postural control variables derived from CoP signals, including displacement, velocity, and acceleration in the cranio–caudal and medio–lateral directions [58,62,63,64,66]. No included studies applied nonlinear analysis to electromyographic signals as the primary data source.

Regarding the relationship between context and instrumentation, laboratory-based studies predominantly used video cameras [43,44,60,65], optoelectronic motion capture systems [23], or force platforms [66]. In contrast, hospital and home-based studies primarily relied on pressure-sensitive mats [58,62], accelerometers [56,57,59,61], and wearable sensors or smartphone-based systems [45,67,68,69]. Notably, the writhing movement phase was almost exclusively assessed using video-based or optoelectronic systems, with only two studies employing pressure-sensitive mats [58,63] and none using force platforms during this developmental period.

Sampling frequencies also showed considerable heterogeneity across acquisition systems and studies. Acquisition rates ranged from 5 Hz in studies using pressure-sensitive mats [58,62,63,64] to 1000 Hz in the force-platform study [66]. IMU-based studies generally employed sampling frequencies of approximately 20 Hz [45,68], whereas video-based and pose-estimation approaches reported frequencies between 24 and 60 Hz [43,44,60,65,69].

### 3.5. Data Collection Protocol

In almost all the included studies, data acquisition was conducted under highly comparable baseline conditions. Infants were consistently positioned supine during an active waking or quiet alert state, typically undressed or wearing only a diaper, in a calm environment without external stimulation or task demands [23,43,44,56,57,58,59,60,61,62,63,64,65,66,67,69]. Two studies were exceptions, as they assessed infants in naturalistic daily life conditions, where positioning was not restricted to the supine posture [45,68].

Despite this consistency in positioning and behavioural state, substantial variability was observed in data collection duration across studies. In laboratory-based settings, recording durations ranged from approximately 2 to 34 min per session [23,43,44,60,65,66]. In studies conducted exclusively in hospital settings or across combined hospital–home contexts, data collection durations varied widely, ranging from 3 to 5 min [56,57,58,59,61,62,63,64,67,69] to extended recordings lasting up to 8 h per day, enabling the analysis of spontaneous movements across prolonged periods of natural daily activity [45,68].

Among the six studies conducted in home settings [45,56,58,59,62,68], studies employing wearable inertial sensors reported the longest data collection durations [45,68]. However, these investigations focused exclusively on specific body segments, such as the right wrist or the lower limbs, and predominantly included samples from infants with specific characteristics such as neurological or developmental risk or indigenous infants. This highlights a trade-off between recording duration, ecological validity, and the spatial scope of movement analysis across different acquisition protocols.

### 3.6. Nonlinear Measures

Across the included studies, a broad range of nonlinear analytical techniques was applied to characterise the temporal structure, complexity, and organisation of spontaneous infant movements. These techniques were applied to movement-derived kinematic and kinetic signals and can be grouped into five main methodological families: entropy-based measures, state-space and dynamical systems measures, recurrence-based analyses, symbolic or discrete-state approaches, and variance- and frequency-based nonlinear descriptors (Appendix A.2).

#### 3.6.1. Entropy-Based Measures

Entropy-based measures were the most frequently applied nonlinear techniques across the included studies. ApEn was predominantly used in studies focusing on early postural control, particularly through center of pressure (CoP) displacement signals recorded during spontaneous supine movements [58,60,62,63,64]. Across these studies, ApEn was applied using a consistent parameterisation, with an embedding dimension of m = 2, a tolerance threshold of r = 0.2 × the standard deviation of the signal, and fixed time-series lengths (typically 500 samples at 5 Hz). This methodological consistency enabled comparisons across developmental stages and between infant groups within and across longitudinal cohorts.

More recent studies increasingly employed Sample Entropy (SampEn) to quantify the regularity and complexity of spontaneous movements derived from kinematic signals, including joint angles, joint angular velocities, limb accelerations, and CoP-derived variables variables [45,66,67,69]. Although parameter choices varied across studies, SampEn was commonly computed using low embedding dimensions (m = 2 or 3) and tolerance values defined as a proportion of the signal standard deviation and was applied to time series ranging from short laboratory recordings to extended home-based datasets.

In addition, one recent study applied FuzzyEn to quantify the complexity of spontaneous lower-limb movements recorded using wearable inertial sensors during daily activities [68]. FuzzyEn was calculated from peak acceleration time series using standard entropy parameters (m = 2, r = 0.2) and a fuzzy membership function, allowing graded similarity between signal patterns. This approach was selected to improve robustness to noise and to accommodate the variability inherent to long-duration, naturalistic recordings. Higher FuzzyEn values were significantly associated with better motor outcomes at 12 months of age, supporting the sensitivity of entropy-based metrics to clinically meaningful aspects of spontaneous movement organisation.

#### 3.6.2. State-Space and Dynamical Systems Measures

Several studies applied state-space reconstruction and dynamical systems approaches to investigate the underlying dynamics of spontaneous infant movements. These included estimation of the embedding dimension using the False Nearest Neighbours (FNN) method, calculation of the Largest Lyapunov Exponent (LyE), and analysis of Mutual Information (MI) to inform time-delay selection or assess interlimb coupling [56,57,59,61]. Across these studies, time delays were typically selected based on the first minimum of the mutual information function or the first zero of the autocorrelation function (approximately 250 ms), and embedding dimensions were determined empirically using FNN criteria. LyE was estimated using standard algorithms (e.g., the Kantz method) with fixed embedding parameters within each study. These measures were primarily applied to triaxial linear acceleration signals recorded from the upper or lower extremities.

#### 3.6.3. Recurrence-Based Analysis

Recurrence-based approaches were employed to examine the temporal recurrence and stability of movement patterns during spontaneous activity. Recurrence Plot (RP) and Recurrence Quantification Analysis (RQA) were applied to multidimensional joint angle time series or acceleration-derived state-space trajectories [43,44,56]. RQA was performed using predefined embedding dimensions and recurrence thresholds, with parameters held constant within each study. Extracted RQA metrics included recurrence rate, determinism, laminarity, trapping time, and entropy, enabling quantification of repeated configurations, intermittent stabilisation phases, and transitions between movement states.

In several studies, recurrence-based analyses were complemented by surrogate data testing to assess whether the observed dynamics reflected deterministic rather than stochastic processes [56,57].

#### 3.6.4. Symbolic and Discrete-State Approaches

Symbolic and discrete-state approaches were used to reduce continuous movement signals into categorical representations, enabling the analysis of configuration patterns and state probabilities. Symbolic dynamics were applied to joint kinematic data or spatial limb relationships by discretising limb positions according to predefined angular or spatial thresholds, generating a finite set of possible movement configurations [54,65]. Symbolic sequences were subsequently analysed to quantify configuration frequency, duration, recurrence, and transitions between states.

More recent studies applied symbolic barcoding approaches to wearable sensor data, generating discrete state sequences from lower-limb acceleration and angular velocity signals and combining these representations with entropy-based metrics [45]. These approaches enabled the characterisation of movement diversity and state distribution during spontaneous activity in ecologically valid contexts.

#### 3.6.5. Variance- and Frequency-Based Nonlinear Descriptors

Hjorth parameters (Activity, Mobility, and Complexity) were applied in one study to velocity time series of upper and lower limb movements during the fidgety period [23]. These parameters were computed within sliding windows and averaged across limbs and spatial components, providing complementary descriptors of signal variance, dominant frequency content, and temporal organisation.

**Figure 3:**
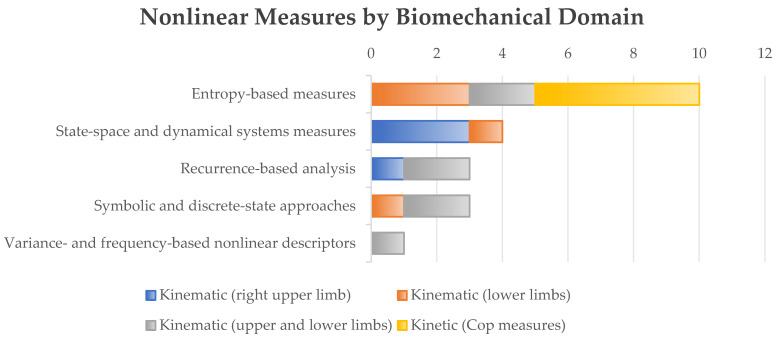
Distribution of nonlinear measures across biomechanical domains: right upper-limb kinematics, lower-limb kinematics, upper and lower limbs kinematics, and kinetics.

Figure 3 shows the distribution of the number of included studies reporting nonlinear measures across biomechanical domains (kinematic or kinetic).

## 4. Discussion

The objective of this review was to identify and synthesise the nonlinear approaches applied to the assessment of spontaneous movements in infants up to 6 months of age, while mapping participant characteristics, assessment contexts, instruments, data collection protocols, and the main variables analysed. By providing an integrated overview of existing methodologies, this review aims to contribute to a clearer understanding of early motor complexity and to inform future efforts toward methodological harmonisation, which may ultimately support more consistent and clinically meaningful assessment practices.

### 4.1. Interpretation of Participants Characteristics 

The heterogeneity observed across the samples of the included studies reflects a well-recognised challenge in paediatric and developmental research, particularly during early infancy, namely difficulties related to recruitment, retention, and standardisation of protocols across clinical and laboratory settings [4,6]. The predominance of small sample sizes limits the generalisability of findings and complicates comparisons between studies, although it also allows for detailed and controlled analyses of movement behaviour [43,44,56]. This duality highlights the persistent need to balance methodological rigour with sample representativeness in future investigations.

Some studies focused on healthy full-term infants, reflecting a clear interest in identifying normative patterns of spontaneous movements that may serve as reference frameworks for comparison with clinical populations [4,9,23,43,44,56,59,62,65,66,68]. While this approach is methodologically valuable, many of these studies were exploratory in nature and relied on relatively small samples.

Even studies including larger samples, such as Oh et al. [68], investigated highly specific populations and movement features, thereby limiting the generalisability of their findings to broader infant populations.

In contrast, the inclusion of preterm infants, infants at neurological risk, or those with specific pathologies underscores the clinical relevance of this field, as these populations are particularly vulnerable to alterations in motor development [45,57,58,60,61,63,64,67,69,70,71,72]. This diversity enables the investigation of distinct developmental trajectories but also increases the complexity of synthesising findings, given that baseline neurological and developmental conditions strongly influence movement variability and organisation [12,22,23,73,74].

Regarding age at assessment, the included studies collectively covered critical periods of early motor development. Several studies focused primarily on the writhing movement period [43,44,56,57,58,67,69], while others specifically targeted the fidgety movement period [23,64,66]. Longitudinal investigations followed the transition between these phases, capturing developmental changes across early infancy [59,60,61,62,63,65,68]. Given that the evolution of spontaneous movements during the first months of life is a sensitive marker of neurological development [4,5,6,9,11,73], this temporal coverage represents a major strength of the existing literature. However, the inclusion of studies assessing infants across wide and heterogeneous age ranges, sometimes encompassing voluntary movements, introduces additional variability that must be considered cautiously when interpreting findings [45].

Overall, the diversity of participant characteristics reinforces both the scientific and clinical relevance of spontaneous movement analysis, while simultaneously highlighting the need for clearer definition of reference groups and harmonisation of assessment windows.

Addressing these issues may enhance comparability across studies and support the development of more robust reference frameworks for future clinical translation.

### 4.2. Interpretation of Context and Data Collection Protocol

The diversity of assessment contexts identified in this review reflects differing methodological priorities, as well as practical and logistical constraints inherent to early infancy research. Laboratory-based studies were typically characterised by smaller samples and a predominance of healthy full-term infants assessed under highly controlled conditions [23,43,44,60,65,66], enabling the detailed characterization of movement dynamics but limiting generalizability. In contrast, studies conducted in hospital and home settings often included larger and at-risk samples, such as preterm or neurologically impaired infants [45,57,58,61,62,63,64,67,68,69], with data collection in these settings facilitating longitudinal follow-up and capturing movements in more natural-istic environments, thereby enhancing ecological validity [45,68].

Across studies, recording durations varied substantially, ranging from short seg-ments of 3–5 min, commonly used in laboratory or clinical settings, to extended re-cordings lasting several hours per day in home-based protocols. While shorter record-ings are easier to implement and reduce the likelihood of external interference, they may not fully capture the temporal variability and intermittency characteristic of spontaneous infant movements. Conversely, prolonged recordings provide a more comprehensive representation of motor dynamics but pose additional challenges re-garding data management and signal processing [45].

Despite the heterogeneity in assessment contexts and recording durations, a high degree of methodological consistency was observed with respect to infant positioning and behavioural state. Most studies assessed infants in the supine position, undressed or wearing only a diaper, during active wakefulness and in calm environments without external stimulation [23,43,44,56,57,58,59,60,61,62,63,64,65,66,67,69]. This uniformity represents a clear methodo-logical strength, as it reduces contextual confounding and supports comparability across investigations. However, careful consideration is required when evaluating studies that investigate spontaneous movement in different postures [45,68].

Overall, the diversity of assessment contexts observed in the literature highlights an inherent tension between experimental control and ecological validity. While la-boratory-based protocols offer greater standardisation and analytical precision, hos-pital and home environments more closely reflect the clinical and everyday realities of infant behaviour. Future studies should seek to balance methodological control with ecological validity to improve the relevance and applicability of spontaneous move-ment assessment protocols [4,6,75].

### 4.3. Interpretation of Instruments and Variables

The wide range of instruments identified across the included studies reflects not only the absence of consensus regarding optimal assessment methodologies, but also the need to adapt data acquisition strategies to different contexts, populations, and analytical aims. Acquisition systems also differed substantially in their technical char-acteristics, including sampling frequency and signal type, factors that may influence the validity, interpretation, and comparability of nonlinear analyses. Importantly, the choice of instrument directly constrained the type of movement-derived variables that could be extracted and, consequently, the nonlinear measures applied and their physi-ological interpretability. In laboratory settings, video-based, optoelectronic, and force platform systems predominated, enabling high spatial precision and rigorous experi-mental control [23,43,44,60,65,66]. These systems supported the extraction of detailed kinematic and kinetic variables, including joint angles, angular velocities, limb trajec-tories, inter-limb spatial relationships, whole-body configurations, and centre of pres-sure displacement and derivatives. However, these approaches are logistically de-manding, costly, and time-intensive, often resulting in smaller samples and limiting broader applicability [76].

In hospital and home settings, greater use was observed of pressure-sensitive platforms, accelerometers, and wearable sensors [45,56,57,58,59,61,62,68]. These technolo-gies are less intrusive and more compatible with clinical workflows and family rou-tines, facilitating the inclusion of at-risk populations and longer recording durations [77]. They provide continuous time series of linear acceleration and, in some cases, angular velocity, enabling prolonged monitoring of spontaneous movements in natu-ralistic environments. Nevertheless, this gain in ecological validity often comes at the expense of reduced spatial specificity, which constrains the range of kinematic variables that can be derived and limits the analysis of segmental or joint-level coordination [56,58,76].

An important observation concerns the assessment of writhing movements, which were almost exclusively evaluated using video-based or optoelectronic systems, with only two studies employing pressure-sensitive platforms and none using force plat-forms during this developmental period [58,63]. As noted by Dusing et al. [58], pressure platforms present inherent limitations compared with force platforms, including the inability to measure shear forces and reduced spatial accuracy. These constraints may affect the robustness and interpretability of kinetic data in supine infants and may partly explain the preference for kinematic-based nonlinear analyses during the writhing movement phase.

The emergence of wearable inertial sensors represents a significant methodological advance, enabling continuous monitoring of spontaneous movements over extended periods, in some cases across multiple days [45,68,77,78]. These technologies may capture behavioural fluctuations across naturalistic environments and therefore provide richer temporal information regarding movement dynamics. However, the management and processing of large volumes of heterogeneous data remain important methodological challenges [79].

Overall, instrument selection across studies reflects a balance between methodological precision and practical feasibility. Laboratory-based systems offer high accuracy and detailed spatial information, whereas portable and less intrusive technologies are better suited to clinical and home-based monitoring and facilitate the inclusion of vulnerable populations. Although this methodological diversity enriches the field, it also highlights the need for greater consistency in reporting acquisition protocols and variable selection to improve comparability across studies [4,6].

### 4.4. Interpretation of Nonlinear Measures

The nonlinear measures identified in this review reflect a conceptual shift from describing spontaneous infant movements in terms of magnitude or frequency toward understanding their temporal organisation, adaptability, and self-organisation [73,74]. Across studies, nonlinear approaches were not used interchangeably; instead, their selection depended on the type of signal analysed (e.g., kinematic trajectories versus center of pressure data), the developmental stage under investigation, and the underlying theoretical perspective on early motor control [43,44,56,58]. This diversity highlights both the richness of the field and the current lack of methodological convergence [4,6].

From a motor control perspective, each methodological family targets a distinct theoretical construct. Entropy-based measures—ApEn, SampEn, and FuzzyEn—index the temporal irregularity and unpredictability of movement, operationalising complexity as the degree to which a system avoids repetitive patterns [40,80]. State-space approaches—embedding dimension and LyE—address the stability and chaotic structure of the underlying attractor, speaking to the degrees of freedom available to the motor system rather than to signal irregularity [49]. Recurrence-based analyses capture the temporal persistence of movement states and the tendency to revisit preferred configurations [81]. Symbolic approaches address the categorical diversity of discrete movement states [82], and Hjorth parameters describe signal variance and dominant frequency content [23]. These distinctions matter for interpretation: studies reporting lower SampEn and studies reporting altered LyE are making fundamentally different claims about motor organisation, even when both are described as reflecting reduced complexity [47,74,80].

The predominance of entropy-based measures, particularly ApEn and SampEn, suggests a strong emphasis on quantifying movement irregularity and temporal un-predictability as proxies for motor complexity [40,83,84]. In studies focusing on early postural control, ApEn was applied in a highly standardised manner to CoP displace-ment signals, allowing longitudinal comparisons across developmental stages and between infant groups [58,62,63,64]. Importantly, these studies demonstrated that en-tropy-based metrics captured changes in temporal structure that were not reflected in traditional magnitude-based variability measures, reinforcing the conceptual distinc-tion between variability and complexity in early motor behaviour [62,63,73]. This supports the view that nonlinear metrics provide complementary information regard-ing motor organisation rather than simply alternative representations of signal am-plitude [80].

More recent investigations have increasingly adopted SampEn to characterise spontaneous movements derived from multi-joint kinematics and wearable sensor data [45,67,69]. Although this shift reflects advances in acquisition technologies and broader data availability, it has also introduced substantial heterogeneity in parameterisation and recording protocols, limiting the direct comparability of entropy estimates across studies. SampEn estimates the probability that two sequences of m consecutive data points that match within a tolerance r will also match at the next point, and its statistical reliability is fundamentally dependent on the number of template matches identified within the time series. Short time series (e.g., 500 samples at 5 Hz) provide fewer template matches, resulting in greater estimation variance and reduced sensitivity to subtle temporal differences [58,62,63,64]. Conversely, prolonged recordings comprising approximately 10^4^ samples may violate stationarity assumptions because behavioural states and movement characteristics are unlikely to remain constant throughout the recording period [45]. Consequently, SampEn values derived from markedly different time-series lengths should not be interpreted as directly comparable, as they may reflect different statistical regimes rather than equivalent representations of movement complexity [85,86]. The tolerance parameter r, commonly fixed at 0.2 × SD of the signal, partially normalises for amplitude differences but does not correct for the dependency of entropy estimates on series length. Therefore, methodological differences in data length and recording conditions may be erroneously interpreted as biological differences in movement organisation [85,86]. In this context, the use of FuzzyEn represents a relevant methodological extension, as it allows graded similarity between patterns and may improve robustness when analysing noisy or long-duration recordings in naturalistic environments [87].

State-space and dynamical systems approaches, including embedding dimension estimation, LyE, and MI, provided a complementary perspective by explicitly addressing determinism, stability, and dimensionality of spontaneous movement dynamics [56,57,59,61]. Across studies, these measures consistently supported the interpretation of infant spontaneous movements as deterministic, chaotic systems rather than random processes. Differences observed between typically developing infants and those with brain injury or developmental risk were interpreted as reflecting altered degrees of freedom, reduced coordination, or diminished adaptive capacity [57,61]. However, these approaches rely on mathematical assumptions that are particularly difficult to satisfy in spontaneous infant movement recordings. Reliable estimation of embedding dimension and LyE requires sufficiently long and relatively stationary time series to permit adequate reconstruction of the underlying attractor. Spontaneous infant movements are, however, inherently intermittent and characterised by bursting activity—discrete episodes of limb motion separated by periods of relative quiescence—rather than the continuous, ergodic dynamics assumed by most state-space reconstruction algorithms. Consequently, the effective number of observations reflecting a coherent dynamical regime may be substantially smaller than the total recording length. Without segmentation or stationarity assessment, LyE estimates may represent the superposition of multiple dynamical regimes rather than a single coherent movement organisation. Notably, none of the included studies employing these approaches reported stationarity testing or burst-detection procedures, limiting confidence in the interpretation of the reported estimates [56,57,59,61]. Conceptually, these approaches target aspects of motor control that are distinct from entropy-based irregularity, namely the stability and structure of the underlying dynamical system, and this distinction reinforces the need to apply them within their valid mathematical domain rather than as interchangeable indices of complexity [49,74].

Recurrence-based analyses further contributed to this framework by revealing intermittent stabilisation around preferred configurations and transitions between ex-ploratory and more constrained movement states [43,44]. Rather than describing con-tinuous variability, RP and RQA highlighted the organisation of spontaneous move-ments around reference states, supporting the notion of hierarchical and self-organised motor control early in life [81]. From a theoretical perspective, such patterns are con-sistent with dynamical systems accounts of motor behaviour, in which stability emerges around preferred states while allowing flexible transitions between explora-tory and constrained modes of coordination [73,74]. The frequent use of surrogate data testing alongside recurrence and entropy measures strengthened the interpretation of spontaneous movements as structured and deterministic, providing methodological validation that extends beyond descriptive complexity metrics [56,57,58,60].

Symbolic and discrete-state approaches offered yet another analytical perspective by transforming continuous movement signals into categorical representations of configurations or states [43,45,65]. These methods revealed that a limited subset of configurations accounts for a large proportion of spontaneous movement behaviour, indicating the presence of preferred organisational patterns even during early infancy [43,73]. More recent symbolic barcoding approaches applied to wearable sensor data extended this logic to ecologically valid contexts, demonstrating reduced state diversity and altered organisation in infants at developmental risk [45]. Collectively, these find-ings highlight the potential of symbolic methods to bridge qualitative descriptions of spontaneous movements with quantitative nonlinear analysis [82].

Finally, variance- and frequency-based nonlinear descriptors, such as Hjorth pa-rameters, were rarely applied but provided complementary information regarding signal variance, dominant frequency characteristics, and temporal organisation. Alt-hough these measures do not quantify complexity in the same sense as entropy-based metrics, they capture nonlinear properties of movement signals that may be particu-larly relevant during transitional developmental phases, such as the fidgety period [23].

Taken together, the diversity of nonlinear measures identified reflects differing conceptualisations of motor variability, including complexity, stability, adaptability, and organization [80]. While each methodological family captures distinct aspects of spontaneous movement behaviour, the predominance of single-measure approaches limits integrative interpretation. Recent methodological perspectives emphasise that nonlinear measures are not interchangeable and should be selected based on explicit theoretical rationale rather than analytical convenience [47,80].

The findings of this review support the adoption of integrative analytical frame-works that combine complementary nonlinear measures and align metric selection with clearly defined motor control constructs. Such integration may facilitate the in-terpretation and future clinical translation of nonlinear movement metrics.

### 4.5. Interaction Between Data Acquisition Technologies, Signal Stationarity, and the Validity of Nonlinear Metrics

The present review has mapped the nonlinear measures applied across studies and catalogued the acquisition systems employed. However, a critical dimension that warrants explicit discussion is the interaction between data acquisition technologies and the mathematical assumptions underlying nonlinear metrics. The validity and in-terpretability of measures such as SampEn, ApEn, and LyE are influenced by factors including signal stationarity, time-series length, sampling frequency, and sig-nal-to-noise ratio. These properties are directly shaped by the acquisition technology used, and failure to account for this dependency may compromise the interpretability and comparability of reported findings.

However, the application of deep learning-based pose estimation to infant movement analysis introduces specific technical limitations that warrant acknowl-edgement. Automated skeletal tracking models such as AlphaPose, employed in the included studies [67,69], estimate joint positions from video frames using probabilistic confidence scores. When confidence thresholds are set too liberally, low-quality joint estimates are retained in the time series; when set too conservatively, tracking gaps introduce discontinuities that require interpolation or exclusion. Both situations in-troduce artificial noise that does not reflect true limb kinematics. Furthermore, deep learning models frequently incorporate temporal smoothing mechanisms that reduce frame-to-frame variability in joint position estimates, effectively regularising the signal prior to nonlinear analysis. Consequently, entropy-based metrics such as SampEn may systematically underestimate movement complexity, as the artificially smoothed signal appears more regular than the underlying movement itself. Skeletal tracking jitter—rapid, low-amplitude oscillations in estimated joint positions arising from model uncertainty rather than actual movement—further contaminates the time series with structured noise that may be erroneously interpreted as movement complexity. Col-lectively, these factors introduce technology-dependent sources of bias that may partly explain differences in nonlinear metric values reported across studies employing different acquisition systems. Notably, neither of the included studies using pose esti-mation [67,69] reported confidence threshold settings, jitter correction procedures, or validation of kinematic outputs against reference motion capture systems, representing an important limitation for the interpretation of the reported nonlinear estimates.

IMUs and triaxial accelerometers, while well suited for long-duration recordings in naturalistic settings [45,56,57,59,61,68], are susceptible to skin-motion artefacts, par-ticularly during periods of high-amplitude or rapid limb movement. These artefacts introduce high-frequency noise into the acceleration time series that does not neces-sarily reflect true limb dynamics. Such artefacts may influence nonlinear metrics that depend on attractor reconstruction and fine temporal structure, including the LyE. Moreover, low sampling frequencies may constrain state-space reconstruction and reduce the reliability of dynamical systems analyses.

Pressure-sensitive mats, used in several studies to derive centre of pressure dis-placement [58,62,63,64], typically operate at sampling frequencies as low as 5 Hz. At this resolution, entropy estimation may be compromised by the limited number of available data points. Although parameter consistency partially mitigates variability, it does not overcome the limitations imposed by low temporal resolution.

A closely related methodological concern pertains to signal stationarity and tem-poral consistency, which influence the validity and interpretation of many nonlinear metrics. Entropy-based measures such as ApEn and SampEn, as well as dynamical systems approaches such as LyE, generally perform best when the statistical properties of the time series—including mean, variance, and temporal structure—remain rela-tively stable throughout the recording window. Although recurrence-based approaches are often considered more robust to non-stationary behaviour, substantial changes in signal characteristics may still complicate the interpretation of recurrence measures.

These assumptions are more likely to be satisfied in short laboratory recordings than in prolonged naturalistic recordings lasting several hours per day [45,68]. Over such intervals, infants inevitably transition across multiple behavioural states—including sleep, feeding, crying, and active wakefulness—each associated with distinct movement dynamics. Consequently, aggregated nonlinear estimates may conflate distinct motor regimes and become difficult to interpret.

Furthermore, longitudinal studies spanning the writhing and fidgety movement phases [59,60,61,62,63,65,68] introduce an additional layer of non-stationarity, as the underly-ing organisation of spontaneous movement is itself undergoing developmental reor-ganisation. In this context, LyE estimates may reflect the superposition of multiple dynamical regimes rather than a single coherent movement organisation. Recurrence measures may also become more difficult to interpret when developmental transitions occur within the analysed time series. Although some studies employed segmented analyses [65], explicit reporting of stationarity assessment procedures remained un-common, limiting confidence in the interpretation of reported nonlinear estimates.

A further dimension of methodological inconsistency concerns the reporting and selection of input parameters for nonlinear metrics-specifically embedding dimension (m), tolerance (r), and time delay (τ)-in relation to the sampling frequencies employed during data acquisition. Across the included studies, these parameters were frequently reported as fixed numerical values without explicit justification of their appropriateness relative to the temporal resolution of the signal. This issue is particularly consequential for the time delay τ, which is expressed in samples rather than absolute time units. A time delay of τ = 1 sample corresponds to 200 ms at a sampling frequency of 5 Hz, but only 5 ms at 200 Hz-a forty-fold difference in the temporal scale of the reconstructed state space. When τ values are reported without reference to sampling frequency, cross-study comparison of dynamical systems parameters becomes fundamentally ambiguous.

Similarly, the embedding dimension m determines, in part, the minimum time-series length required for reliable metric estimation. At low sampling frequencies, the number of available samples may be insufficient to satisfy these requirements, further limiting analytical validity. While the tolerance parameter r, commonly set at 0.2 × SD of the signal, is relatively robust to differences in sampling frequency, it remains sensitive to noise characteristics introduced by different acquisition technologies. Therefore, parameter values cannot be interpreted independently of the temporal resolution at which they were derived.

Collectively, these considerations suggest that differences in nonlinear metric values reported across studies may reflect, at least in part, technology-dependent signal characteristics in addition to genuine differences in movement organisation. Consequently, validation of nonlinear metrics against reference standards remains essential to ensure confidence in the interpretation and comparison of findings.

### 4.6. Limitations of the Review and Available Evidence

Despite the growing interest in nonlinear analysis of spontaneous infant movements, several methodological and conceptual limitations emerged across the included studies. Substantial heterogeneity was observed in sample characteristics, acquisition systems, recording durations, signal types, and analytical parameters. While this diversity reflects the exploratory nature of the field, it limits direct comparability between studies and constrains the generalisability of findings.

Sample size and composition constitute an additional consideration. Many studies relied on small or highly specific samples, often focusing on healthy full-term infants or narrowly defined clinical populations. Although suitable for targeted investigations, this restricts cross-study comparability and limits the extrapolation of findings to more diverse populations. In addition, the use of repeated observations from overlapping cohorts, while methodologically sound, reduces the effective variability represented in the literature.

Another important limitation of the included studies is the frequent reliance on single nonlinear measures. Given that entropy-based metrics, state-space approaches, recurrence analyses, and symbolic methods capture distinct properties of motor behaviour—such as irregularity, stability, determinism, and organisational structure—single-measure approaches may oversimplify the multidimensional nature of spontaneous movements. This highlights the need for integrative analytical frameworks that align metric selection with clearly defined motor control constructs.

Overall, the available evidence base remains limited and should be interpreted cautiously. Most studies were exploratory, relied on relatively small and heterogeneous samples, and frequently included highly specific populations or repeated observations from overlapping cohorts. Consequently, the current evidence is insufficient to support definitive conclusions regarding the normative behaviour, prognostic value, or clinical applicability of nonlinear metrics in infant spontaneous movement assessment.

The review was restricted to studies published between 2005 and 2025, a period selected to capture the emergence and consolidation of nonlinear analytical approaches in infant movement research. The search was conducted across five major databases using a broad strategy designed to maximise coverage of the field. While this approach aimed to ensure representativeness, it is possible that some relevant studies may not have been identified. The use of Google Scholar as a supplementary source also introduces reproducibility concerns, as its ranking algorithm is dynamic and search results may vary across users, sessions, and time points. Furthermore, search results cannot be exported systematically in the same manner as traditional bibliographic databases. To enhance transparency and reproducibility, future reviews should predefine and report Google Scholar search parameters, including the search date, query terms, sorting method, and maximum screening depth. Although eligibility was restricted to studies published in English, Portuguese, and French, the potential impact of language bias is likely to be limited because English is the predominant language of publication in the field of nonlinear movement science. Nevertheless, relevant studies published in other languages may have been missed, and this limitation should be considered when interpreting the findings of the present review.

In addition, the use of the Boolean operator NOT (EEG OR heart) was intended to exclude records focused primarily on neurophysiological or cardiac signals, which fell outside the scope of the review. However, this exclusion criterion may have inadvertently filtered out multimodal studies in which movement-based nonlinear analysis constituted the primary focus, but physiological co-monitoring was also reported. Consequently, potentially relevant studies may have been missed, and this issue should be considered in future reviews addressing multimodal approaches to infant movement assessment.

Additionally, certain sources of grey literature, including conference abstracts, dissertations, and non-peer-reviewed reports, were excluded. This decision was made to ensure the inclusion of studies providing sufficient methodological detail on acquisition systems, preprocessing procedures, and nonlinear parameter settings, which are essential for the interpretation and synthesis of nonlinear analyses. Nevertheless, the exclusion of these sources may have resulted in the omission of relevant emerging evidence and should therefore be considered when interpreting the findings of this review.

### 4.7. Clinical Implications and Future Directions

From a clinical perspective, the findings of this review underscore both the promise and the current constraints of nonlinear movement analysis. Nonlinear metrics consistently demonstrated sensitivity to subtle alterations in movement organisation not captured by traditional linear measures, particularly in infants at neurological risk. These findings suggest that some nonlinear metrics may hold promise for the early identification of atypical motor development. However, this promise should not be interpreted as evidence of established clinical utility. To date, none of the identified nonlinear measures has been validated for routine clinical use, and their application remains confined to research and exploratory settings. Among the available metrics, ApEn and SampEn currently appear to be the most readily translatable to future clinical practice, primarily because they are the most extensively investigated measures and have repeatedly demonstrated sensitivity to developmental differences and neurological risk. However, this should not be interpreted as evidence of superiority over other nonlinear approaches. Recurrence-based and symbolic methods may offer important theoretical and methodological advantages for the analysis of spontaneous infant movements, particularly given their potential robustness to intermittency and non-stationarity. Nevertheless, these approaches remain less extensively studied and currently lack sufficient evidence, standardisation, and validation to support their routine clinical implementation.

Future research should prioritise methodological harmonisation and theoretical clarity through the adoption of consensus-based recommendations regarding acquisition protocols, signal preprocessing, stationarity assessment, parameter selection, and reporting practices. Studies should incorporate formal assessment of signal stationarity, appropriate segmentation procedures, and entropy estimation strategies, while systematically reporting acquisition specifications, preprocessing pipelines, filtering procedures, and parameter-selection strategies, as these factors directly influence metric validity and cross-study comparability.

For entropy-based measures, studies should ensure sufficient data length to obtain reliable template matching and should explicitly justify parameter choices and recording durations [83,84]. Sampling frequency should be selected according to the analytical objectives and explicitly considered when interpreting entropy and dynamical systems measures. For FuzzyEn, reporting of fuzzy membership function parameters remains essential for reproducibility [85].

State-space approaches require sufficiently long and relatively stationary recordings, and future studies should explicitly justify data-length requirements and stationarity assessment procedures. Given the intermittent nature of spontaneous infant movements, segmentation into active movement epochs may improve the validity of state-space analyses. Parameters such as τ should be reported in absolute temporal units to facilitate cross-study comparisons.

For recurrence analyses, embedding parameters and threshold definitions should be transparently justified and consistently reported [86].

Across all metrics, future studies should adopt minimum reporting standards encompassing acquisition specifications, preprocessing procedures, stationarity assessment, parameter settings, and quality-control procedures for emerging technologies such as pose-estimation systems. Multicentre collaborations and shared datasets will be essential for validating these recommendations and developing normative benchmarks. Longitudinal designs linking early nonlinear metrics to later functional outcomes will be critical for establishing prognostic value and supporting clinical translation.

Finally, the increasing use of wearable inertial sensors and video-based pose-estimation systems offers new opportunities for large-scale and ecologically valid data collection. However, the fidelity of signals acquired using these technologies requires further validation before assuming that they necessarily provide richer temporal information for nonlinear analyses. From a practical perspective, ApEn and SampEn currently appear to be the most feasible nonlinear measures for future clinical implementation. Their feasibility stems from their relative computational simplicity, extensive use in the literature, and successful application across a range of acquisition modalities, including wearable sensors and pressure-sensitive devices. Importantly, this practical feasibility should not be interpreted as evidence that all acquisition systems provide equally valid signals for entropy estimation. Rather, further validation of signal quality and acquisition protocols remains essential.

FuzzyEn may also represent a promising alternative because of its greater robustness to noisy recordings. In contrast, state-space approaches such as the LyE require longer and more stationary time series, as well as greater computational expertise, which may limit their immediate applicability in routine clinical settings.

In summary, nonlinear analysis of spontaneous infant movements offers a promising framework for characterising early motor organisation and detecting subtle alterations in movement behaviour that may not be captured by traditional linear approaches. Nevertheless, meaningful clinical translation will depend on methodological harmonisation, transparent reporting standards, normative datasets, and robust longitudinal evidence demonstrating prognostic and clinical utility.

## 5. Conclusions

This scoping review provides a comprehensive overview of the current research landscape on the application of nonlinear measures to the analysis of spontaneous movements in infants during the first six months of life. Entropy-based measures, particularly ApEn and SamEn, were the most frequently applied techniques to characterise movement complexity, reflecting a predominant interest in quantifying temporal irregularity and organisation beyond traditional linear descriptors.

The analysis focused mainly on spontaneous movements assessed in the supine position, encompassing both writhing and fidgety movements. Kinematic variables derived from limb and joint motion, as well as kinetic variables related to CoP displacement, were the most analysed signals. These variables were primarily obtained using video-based systems, optoelectronic motion capture, pressure-sensitive platforms, and, more recently, wearable inertial sensors and video-based pose estimation approaches.

Collectively, these nonlinear methods revealed that spontaneous infant movements exhibit structured, deterministic, and self-organised dynamics, with differences observed between typically developing infants and those at neurological or developmental risk.

Despite the methodological diversity identified, the current body of evidence remains preliminary, exploratory in nature, and limited by the predominance of small, heterogeneous, and often clinically specific samples. Consequently, the findings should be interpreted cautiously and cannot yet support definitive conclusions regarding the routine clinical implementation of nonlinear metrics. Meaningful clinical translation will require the development of standardised methodological frameworks, including consensus regarding acquisition protocols, minimum sampling requirements, signal preprocessing procedures, stationarity assessment, and the reporting of metric-specific parameters. Future studies should prioritise multicentre collaborations, shared datasets, and longitudinal designs linking nonlinear metrics to clinically meaningful developmental outcomes. At present, nonlinear measures should be regarded as complementary tools that may augment established observational assessments, such as the General Movement Assessment, rather than as standalone diagnostic markers. Their future clinical value will ultimately depend on robust standardisation and validation processes capable of supporting objective, reproducible, and clinically interpretable assessments of early neurodevelopment.

## Figures and Tables

**Table 1:** Eligibility criteria according to PCC.

	Criteria
Infants up to 6 months of age, with or without risk of neurodevelopmental impairment.	Population
Nonlinear measures applied in the analysis of spontaneous movements in infants.	Concept
Studies conducted in any setting (home-based, clinical or laboratory).	Context

**Table 2:** Characteristics of the studies included in the scoping review.

Author (Year)	Country	Study Design	Participants	Context	Instruments	Sampling Frequency	Protocol	Nonlinear Measures	Main Variables
**Aßmann et al.** **(2006, 2007) [43,44]**	Germany	Exploratory observational (mini-longitudinal)	*N* = 6 (3M; 3F) healthy full-term newborns. Gestational age: 37–39 weeks. Birth weight: 2040 to 3360 g. Age Assessment: between 1 and 10 days old, 2 to 3 times.	Laboratory setting	3 synchronized video cameras Ariel Performance Analysis System (APAS) 3D reconstruction of joint kinematics from synchronized 2D recordings	50 Hz	Infants were undressed, awake, and positioned supine. No external stimuli were presented, and posture was not constrained. Recording duration: 20 min.	SD and RP RQA, RP and SD	Kinematic: Joint angles (°) (shoulder, elbow, hip, knee); hand/foot trajectories. Hand/foot velocities (mm/s). Body configurations. Frequency and duration of configurations. Transition patterns.
**Ohgi et al. (2007) [56]**	Japan	Exploratory observational (cross-sectional)	*N* = 6 (3M; 3F) full-term newborns. Birth weight: 3013.5 g. Age Assessment: 1 month old.	Home setting	Triaxial accelerometer (MVP-A304Ac Micro Stone Co., Japan)	200 Hz	Sensor attached below the right wrist; recording performed during active–alert state, supine position; no manipulation. Recording duration: 200 s.	RP FNN LyE SD (AAFT) PE analysis Mutual information	kinematic Linear Acceleration 3D of the right upper limb (m/s^2^)
**Ohgi et al. (2008) [57]**	Japan	Exploratory observational (cross-sectional)	*N* = 14 *N* = 7 preterm infants with brain injuries (PVL or IVH) (5M; 2F) Gestational Age: 31.1 weeks *N* = 7 matched low-risk preterm infants (5M; 2F) Gestational Age: 30.6 weeks Age Assessment: 1-month post-term (both groups)	Hospital (NICU/outpatient follow-up)	Triaxial accelerometer (MVP-A304Ac; Micro Stone Co., Japan)	200 Hz	Sensor attached below the right wrist; recording performed during active–alert state, supine position; no manipulation. Recording duration: 200 s.	FNN LyE PE analysis Surrogate data analysis (AAFT)	kinematic Linear Acceleration 3D of the right upper limb (m/s^2^)
**Dusing et al. (2009) [58]**	United States of America	Observational (cross-sectional)	*N* = 32 without diagnosed neurological or genetic conditions: *N* = 15 term (3M; 12F) Gestational age: 38.9 ± 1.1 weeks Age Assessment: 41.0 ± 1.1 weeks post-conceptual age *N* = 17 pre-term (10M; 7F) Gestational age: 31.9 ± 3.0 weeks Age Assessment: 41.7 ± 0.7 weeks post-conceptual age.	Home setting	Pressure-sensitive mat (Conformat™), synchronized with digital video.	5 Hz	Infants were positioned supine, undressed, awake and in a quiet alert or active alert behavioral state on a pressure-sensitive mat. Recording duration: 5 min.	ApEn Surrogate data analysis (determinism testing)	Kinematic: Center of pressure (COP) displacement in caudal–cephalic and medial–lateral direction.
**Gima et al. (2011) [59]**	Japan	Observational (longitudinal)	*N* = 8 healthy full-term newborns (3 M; 5 F) Gestational age: 39.0 ± 1.1 weeks Birth weight: 3070.6 ± 287.8 g Age Assessment: every fourth week between birth (0 months) and 6 months.	Home setting	Tri-axial accelerometer (MVP-A304Ac, Micro Stone Co., Japan);	200 Hz	Sensors placed just below the ankles; recording performed during active–alert state, supine position; no manipulation. Recording duration: 200 s.	FNN LyE MI	Kinematic variables: 3D linear acceleration of the right and left lower extremities (m/s^2^)
**Smith et al. (2011) [60]**	USA	Observational (Cross-sectional and longitudinal)	*N* = 31, contributing a total of 56 observations. *N* = 11 infants with myelomeningocele contributing to 26 repeated observations Gestational age: ≥28 weeks. *N* = 20 infants with typical development, contributing for 30 repeated observations Age Assessment: 1, 3, 6, and 9 months.	Laboratory setting	3D motion capture systems: Study 1: Vicon Peak Motus system, 6 cameras Study 2: Two synchronized digital video cameras	60 Hz	Reflective markers attached to the lateral surface of the greater trochanter, ventral surface of the patella and ventral surface of the third metatarsal. Infants were positioned supine, undressed, during active–alert state, no manipulation. For Study 1, infants completed 2 min trials and 1 min trial per session; for Study 2, movements were recorded for 2 min per leg.	ApEn Surrogate data analysis (determinism testing)	Kinematic: Hip sagittal plane angle (°) Frequency of hip flexion/extension events (>2° within 167 ms)
**Gima et al. (2013) [61]**	Japan	Comparative observational (longitudinal)	*N* = 11 *N* = 7 healthy full-term infants (3 M, 4 F) Gestational age: 39.0 ± 0.9 weeks Birth weight: 2990.7 g ± 327.5 g *N* = 4 premature infants with periventricular leukomalacia (*n* = 3) and intraventricular hemorrhage (*n* = 1) (3M, 1F) Gestational age: 30.3 ± 4.8 weeks Birth weight: 1367.5 ± 429.3 g Age Assessment: monthly from 1 to 4 months of corrected age.	Healthy full-term infants assessed during home visits Infants with brain injuries assessed in hospital	Tri-axial accelerometer (MVP-A304Ac)	200 Hz	Sensor attached below the right wrist; recording performed during active–alert state, supine position; no manipulation. Recording duration: 200 s.	FNN LyE	Kinematic: 3D linear acceleration of the right upper extremity (m/s^2^) Resultant acceleration magnitude (m/s^2^)
**Dusing et al. (2013, 2014, 2016)** **[62,63,64]**	USA	Observational (longitudinal)	2013: *N* = 22 healthy full-term infants (11M; 11F) Gestational age: 39.5 ± 1.1weeks Birth weight: 3311 ± 499 g Age Assessment: 0.5–1.5 months, biweekly visits until 3 months and monthly visits from 3 to 6 months. 2014: *N* = 40: *N* = 18 infants born preterm (risk factors: IVH, ventricular shunt, chronic lung disease) (10M; 8F) Gestational age: 28.3 ± 3.1 weeks Birth weight: 1178 ± 493 g *N* = 22 healthy full-term infants (10M; 8F) Same cohort as Dusing et al. (2013) [62] Age Assessment: 0.5–1.5 months, biweekly visits until 3 months and monthly visits from 3 to 6 months of corrected age. 2016: *N* = 18 infants born preterm (10 M; 8 F) Same cohort as Dusing et al. (2014) [63] Age Assessment: Biweekly visits until 3 months and monthly visits from 3 to 6 months of corrected age.	2013: Home-setting 2014/2016 Not explicitly reported (home setting inferred from protocol)	Pressure-sensitive mat (Conformat™), synchronized with digital video.	5 Hz	Infants were positioned supine, undressed, awake and in a quiet alert or active alert behavioral state on a pressure-sensitive mat. Recording duration: 5 min.	ApEn	Kinematic: CoP displacement in caudal–cephalic and medial–lateral direction (mm)
**Aßmann et al. (2019) [65]**	Germany	Exploratory observational (longitudinal)	*N* = 14 healthy full-term infants, contributing recording episodes under different technical conditions: Group 1: *N* = 7 (4F, 3M) Gestational age: 40.2 ± 0.8 weeks Age Assessment: 5.7 ± 6.8 days Group 2: *N* = 2 infants (1M; 1F) Gestational age: 40.0 ± 0.0 weeks Age Assessment: 45.5 ± 22.1 days Group 3: *N* = 5 (1M; 4F) Gestational Age: 39.5 ± 1.0 weeks Age Assessment: 41.4 ± 29.6 days	Laboratory setting (neonatal unit)	Multi-camera video motion capture system 3D reconstruction of hand and foot trajectories	50 Hz	Infants positioned supine, undressed, awake, and allowed to move spontaneously. No external stimulation or task constraints were applied. Recording duration: 4–34 min. Group 1: soft markers on the forehead, chest, shoulders, elbows, hands, hips, knees, and feet. Group 2: Passive reflecting markers placed on the forehead, chest, navel, hands, and feet. Group 3: Passive reflecting markers on the forehead, chest, upper arms, forearms, hands, thighs, calves, and feet.	Symbolic dynamics Symbolic recurrence analysis	Kinematic: Radial distance of hands and feet (mm) Radial and spatial displacement, spatial (mm) Tangential velocity (mm/s)
**Marchi et al. (2020) [23]**	Italy	Exploratory observational (Cross-sectional)	*N* = 8 typically developing, low-risk, full-term infants with normal fidgety general movements (6M; 2F) Gestational age: 39 (36–41) weeks Age Assessment: 9–20 weeks of post-term age	Laboratory setting	Optoelectronic motion capture system (SMART-D, BTS, Italy) 10 infrared cameras Reflective markers on hands, feet, glabella, and pubic symphysis Synchronous video for qualitative scoring	200 Hz	Reflective markers were fixed on the dorsal surface of each hand and foot, on the glabella and on the pubic symphysis. Infants were in supine position, undressed, during active wakefulness, minimizing environmental interference. Recording duration: ≥ 3 min, 3 consecutive times.	Hjorth parameters (Activity, Mobility, Complexity)	**Kinematic:** Inter-limb distances (m and limb height from ground (m) Probabilities of close/lifted limb positions
**Wang et al. (2022) [66]**	United States of America	Observational (cross-sectional)	*N* = 19 healthy full-term infants, classified as normal fidgety movements by GMA (9M; 10F) Gestational Age: >37 weeks Age Assessment: 14.7 ± 4.5 weeks adjusted age Weight: 6200 ± 800 g	Laboratory setting	Force platform (AMTI) GoPro video camera (for GMA scoring)	1000 Hz	Infants were positioned supine on a force platform during the active period, undressed, in a calm environment, with no external stimuli Recording duration: 2 min.	SampEn	Kinetic: CoP (CoP) (mm) displacement CoP velocity (mm/s) CoP acceleration (mm/s^2^)
**Shin et al. (2022) [67]**	Republic of Korea	Observational (Prospective)	*N* = 65 very preterm infants *N* = 16 (8M; 8F) HINE < 60 Gestational age at birth: 28 weeks and 5 days (± 3 weeks and 2 days) Birth weight: 1128.7 ± 421.6 g Age Assessment: 11 weeks 3 days post-natal age *N* = 49 (31M; 18F) HINE ≥ 60 Gestational age at birth: 28 weeks and 5 days (± 2 weeks and 6 days) Birth weight: 1080.3 ± 340.7 g Age Assessment: 11 weeks 4 days post-natal age	Clinical and home-based	Conventional or smartphone RGB video cameras AlphaPose deep learning-based pose estimation model Clinical reference standard: HINE and GMA	Not reported	Infants were positioned supine, awake, calm, and with no external stimulation. Recording duration: 3–5 min. Twelve joint positions (bilateral shoulders, elbows, wrists, hips, knees, and ankles) were automatically extracted using AlphaPose.	SampEn	Kinematic: Joint angles (°) Joint angular velocities (°)
**Deng et al. (2023) [45]**	China	Exploratory observational (Cross-sectional)	N = 20: *N* = 11 with typcal development (8M; 3F) Gestational age: 39.3 ± 1.3 weeks Weight: 7340 ± 143 g Age Assessment: 113.5 ± 73.4 days *N* = 9 at risk (5M; 4F) Gestational age: 31.2 ± 6.4 weeks Age Assessment: 267.44 ± 129.86 days of corrected age.	Home environment	Wearable inertial measurement units (IMUs) (Opal, APDM Inc.).	20 Hz	Sensors are attached bilaterally to the ankles. Spontaneous lower-limb movements were continuously recorded during daily activities in the home environment for two full days (≥ 8 h/day).	SampEn Symbolic dynamics-based barcoding analysis	Kinematic: Linear acceleration (m/s^2^) Angular velocity (rad/s) Movement duration (s) Peak acceleration (m/s^2^) Peak angular velocity (rad/s) State probabilities (dimensionless)
**Oh et al. (2024) [68]**	Guatemala/USA	Observational Longitudinal	*N* = 40 (20M; 20F) healthy full-term infants (indigenous) Gestational age: >38 weeks Birth weight: 3100 g (2800–3500 g) Age Assessment—3 visits: Visit 1: mean 63 days Visit 2: mean 94 days Visit 3: mean 129 days	Home or clinical setting	Wearable inertial measurement units (IMUs) (Opal v2, APDM Inc.).	20 Hz	Sensors attached bilaterally to ankles. Infants followed under natural daily conditions; no task constraints. Recording duration: ~10–12 h/day.	FuzzyEn	Kinematic: Linear acceleration (m/s^2^) Angular velocity (rad/s) Movement rate (movements/hour) Peak acceleration (m/s^2^)
**Park et al. (2024) [69]**	Republic of Korea	(Observational prostective	*N* = 90 very preterm or very low birth weight infants: *N* = 11 with motor developmental delay Gestational Age: 27 weeks 3 days ± 2 weeks 3 days Birth weight: 1029.3 ± 383.0 g Age Assessment: 12 weeks 3 days ± 2 weeks 3 days of post-natal age. *N* = 79 with typical development (53M; 26F) Gestational age: 29 weeks ± 2 weeks and 6 days Birth weight: 1149.2 ± 361.3 g Age Assessment: 11 ± 3 weeks of post-natal age.	Clinical or home-based setting	Smartphone RGB video cameras Deep learning-based pose estimation model (AlphaPose) Clinical reference: GMA and BSID-III (motor domain)	24 Hz	Infants in the supine position, awake, calm, no external stimulation. Recording duration: 3–5 min. Twelve joint positions (bilateral shoulders, elbows, wrists, hips, knees, and ankles) were automatically extracted using AlphaPose.	SampEn	Kinematic: Joint angles (°) of bilateral shoulders, elbows, wrists, hips, knees, and ankles Joint angular velocities (°) of bilateral shoulders, elbows, wrists, hips, knees, and ankles

**Legend:** Values are presented as mean ± standard deviation (SD) or median (range), as reported in the original studies. Sample characteristics are described exactly as provided in the respective publications. When studies include repeated assessments of the same cohort, this is indicated in the Study Design column. Abbreviations used in the table are as follows: AAFT, Amplitude Adjusted Fourier Transform; ApEn, Approximate Entropy; CoP, Center of Pressure; DET, Determinism; FNN, False Nearest Neighbors; FuzzyEn, Fuzzy Entropy; GMA, General Movement Assessment; HINE, Hammersmith Infant Neurological Examination; IVH, Intraventricular Hemorrhage; LAM, Laminarity; LyE, Largest Lyapunov Exponent; MI, Mutual Information; NICU, Neonatal Intensive Care Unit; PE, Prediction Error analysis; PVL, Periventricular Leukomalacia; RP, Recurrence Plot; RQA, Recurrence Quantification Analysis; RR, Recurrence Rate; SampEn, Sample Entropy; SD, Surrogate Data analysis (unless otherwise specified as Symbolic Dynamics); TT, Trapping Time; and ENTR, entropy derived from recurrence quantification analysis. Kinematic variables include joint angles (°), linear acceleration (m/s^2^), angular velocity (rad/s or °/s as reported), tangential velocity (mm/s), spatial displacement (mm), inter-limb distances (m), and movement duration (s). Kinetic variables include center of pressure displacement (mm), velocity (mm/s), and acceleration (mm/s^2^). Entropy-based measures, recurrence indices, symbolic metrics, and probability-based parameters are dimensionless unless otherwise specified. **Supplementary notes:** Aßmann et al. [43,44] applied complementary nonlinear techniques to the same dataset of six healthy full-term neonates, whereas Aßmann et al. [65] extended this approach to a different cohort to examine early developmental trajectories of spontaneous movement. Studies reporting multiple groups present sample characteristics separately by group, and repeated observations in longitudinal designs refer to multiple recording sessions from the same infants. Symbolic dynamics, surrogate data analysis, and power spectrum analysis are reported when used as methodological or supportive analyses. Only movement-based signals were considered eligible. The studies by Dusing et al. [62,63,64] derive from partially overlapping longitudinal cohorts but are presented separately, as each paper addresses a distinct analytical focus.

## Data Availability

Not applicable.

## Abbreviations

The following abbreviations are used in this manuscript:

AAFT	Amplitude Adjusted Fourier Transform
ApEn	Approximate Entropy
CNS	Central Nervous System
CoP	Center of Pressure
DET	Determinism
EEG	Electroencephalography
EMG	Electromyography
ENTR	Entropy Derived From Recurrence Quantification Analysis
FM	Fidgety Movements
FNN	False Nearest Neighbors
FuzzyEn	Fuzzy Entropy
GM	General Movements
GMA	General Movement Assessment
HINE	Hammersmith Infant Neurological Examination
IEEE	Institute of Electrical and Electronics Engineers
IMU	Inertial Measurement Unit
IVH	Intraventricular Hemorrhage
JBI	Joanna Briggs Institute
LAM	Laminarity
LyE	Largest Lyapunov Exponent
MI	Mutual Information
MMC	Myelomeningocele
NICU	Neonatal Intensive Care Unit
OSF	Open Science Framework
PCC	Population-Concept-Context
PE	Prediction Error Analysis
PRISMA-ScR	Preferred Reporting Items for Systematic reviews and Meta-Analyses extension for Scoping Reviews
PVL	Periventricular Leukomalacia
RP	Recurrence Plot
RQA	Recurrence Quantification Analysis
RR	Recurrence Rate
SampEn	Sample Entropy
SM	Spontaneous Movements
SD	Surrogate Data Analysis
TT	Trapping Time
WM	Writhing Movements

## Appendix A

### Appendix A.1. Search Strategy Adapted for All Databases

**PubMed^®^**	(infant* OR newborn OR baby* OR neonate) AND (movement OR “spontaneous movements”) AND (nonlinear* OR “non-linear” OR entropy OR Lyapunov OR “complexity” OR “time series”) NOT (EEG OR heart)
**Institute of Electrical and Electronics Engineers^®^**	(infant* OR newborn OR baby* OR neonate) AND (movement) AND (nonlinear OR “non-linear” OR entropy OR “time series” OR Lyapunov OR complexity) NOT (EEG OR heart)
**Web of Science™**	(Infant* OR newborn OR baby OR neonate) AND (movement OR “spontaneous movements”) AND (nonlinear OR “non-linear” OR entropy OR “time series” OR Lyapunov OR complexity) NOT (EEG OR heart)
**ScienceDirect^®^**	(infants OR newborn) AND (“general movements” OR “spontaneous movements”) AND (nonlinear OR “non-linear” OR entropy OR “time series”) NOT (heart)
**Google Scholar**	(infant* OR newborn OR baby OR neonate) AND (“spontaneous movements” OR “general movements”) AND (“non-linear” OR nonlinear OR entropy OR “time series” OR Lyapunov) -cardiac -heart -EEG

### Appendix A.2. Nonlinear Measures, Analytical Parameters, and Computational Settings Used in the Included Studies

**Study**	**Nonlinear Measure**	**Parameters Used**	**Signal/Domain**	**Key Findings**
**Aßmann et al. (2006, 2007) [43,44].**	Symbolic Dynamics	- Binary encoding of limb positions (arms <60°/≥60°; legs <120°/≥120°) - 16 configurations	3D kinematic data (multi-joint, whole-body)	Limited set of preferred configurations with high recurrence, suggesting reference states
	Recurrence Plot (RP)	- Embedding dimension m = 4; delay τ ≈ 10–20 frames; radius ε ≈ 30 (normalized units); sliding window ≈ 300 samples	3D joint angle time series from 4 limbs	Visual recurrence structures supported the existence of stable configuration states and intermittent transitions.
	Recurrence-based analysis: RP and RQA	- m = 4 - τ ≈ 10–20 frames; - ε ≈ 30 - Sliding window (~300 samples)	Multidimensional joint angle time series	Presence of stable states and intermittent transitions
	RQA indices	- RQA indices extracted from recurrence plots, including: ✓ Recurrence rate (RR) ✓ Determinism (DET) ✓ Laminarity (LAM) ✓ Trapping time (TT) ✓ Entropy (ENTR)	Multidimensional joint angle time series	Evidence of hierarchical and self-organised motor behavior.
**Ohgi et al. (2007) [56]**	FNN	- Delay (τ) = 250 ms (first zero of autocorrelation/first minimum of mutual information) - r = 10	3D linear acceleration time series (x, y, z axes)	Spontaneous upper-limb movements displayed chaotic nonlinear dynamics with optimal embedding dimensions of 5–6, indicating deterministic structure and multiple active degrees of freedom.
	LyE	- Kantz algorithm - m = 5 - Delay (τ) = 250 ms - neighborhood radius ε = 1/10 SD		Positive LyE values across all axes confirm chaotic behavior in newborns’ spontaneous movements.
	MI	- Delay (τ) selected from the first minimum of mutual information (~250 ms)		Confirmed time-dependent structure in spontaneous movement signals and supported appropriate embedding parameter selection.
	RP	- m = 5 - Delay (τ) = 250 ms - Threshold ε small (visual inspection; no RQA metrics)		Recurrence plots revealed intermittent alternations between quiescent and bursting phases, indicating nonstationary and self-organized dynamics.
	Surrogate Data Test	- Amplitude-adjusted Fourier Transform (AAFT); 100 surrogate datasets		Prediction error was significantly lower in original data than in surrogate data (*p* < 0.05), confirming a nonlinear deterministic origin of spontaneous movements.
**Ohgi et al. (2008) [57].**	FNN	Same as 2007	3D linear acceleration time series (x, y, z axes)	Infants with brain injuries exhibited significantly higher embedding dimensions than infants without brain injuries, indicating larger dimensionality and increased degrees of freedom, consistent with reduced organization of motor control.
	LyE	Same as 2007		LyE values were positive in all infants, confirming chaotic dynamics. Infants with brain injuries showed significantly higher LyE values across all axes, reflecting greater instability and unpredictability of spontaneous movements.
	MI	Same as 2007		Confirmed time-dependent structure of spontaneous movement signals and supported consistent embedding parameter selection.
	RP	Same as 2007		Recurrence plots revealed intermittent alternations between quiescent and bursting phases, indicating nonstationary and self-organized movement dynamics.
	Surrogate Data Test	Same as 2007		Prediction errors were significantly lower in original data than in surrogate data for most infants, confirming nonlinear deterministic dynamics. In a subset of infants with brain injuries, surrogate tests failed, suggesting loss of chaotic structure and increased randomness.
**Dusing et al. (2009) [58].**	ApEn	- m = 2; Tolerance r = 0.2 × SD of the signal - Delay (τ) = 1	CoP displacement time series (caudal-cephalic, medial-lateral, and resultant	Infants born preterm exhibited significantly lower ApEn values in the caudal–cephalic direction compared with infants born at full term, indicating more predictable and less complex postural control patterns.
	Surrogate Data Analysis	- Surrogate generation using algorithms described by Theiler et al. - ApEn calculated for original and surrogate time series - Determinism was inferred when original ApEn values were significantly lower than surrogate ApEn values, indicating structured (non-random) temporal organization		Surrogate analysis demonstrated that CoP fluctuations in both preterm and full-term infants were deterministic rather than random, supporting the use of nonlinear analysis to characterize infant postural control.
**Gima et al. (2011) [59].**	FNN	- m determined using FNN method - Delay (τ) = 250 ms (first zero of the autocorrelation function	3D linear acceleration time series (x, y, z axes) of the lower extremities	The optimal embedding dimension was greater than 5 across all ages and limbs, with developmental changes showing a U-shaped pattern (6–7 at birth, decreasing to 5, then increasing again by 6 months), consistent with modulation of degrees of freedom during development.
	LyE	- Kantz algorithm - m = 5; Delay (τ) = 250 ms - Noise reduction applied prior to estimation		Positive LyE values were observed across all ages and limbs (range: 0.79–2.99), confirming chaotic dynamics in spontaneous lower extremity movements throughout early infancy.
	MI	- MI calculated between right and left lower extremity acceleration directions - Low-acceleration periods excluded from analysis		Mutual information was highest at birth and decreased to its lowest values between 3 and 4 months of age, indicating developmental changes in interlimb coordination and coupling.
**Smith et al. (2011) [60].**	ApEn	- m = 2 - Tolerance (r) = 0.2 × SD of the signal - Delay (τ) = 1 sample - Time series length: 6147 samples (102.5 s)	Hip sagittal plane angle time series	Infants with myelomeningocele exhibited significantly lower ApEn values than infants with typical development across all ages, indicating more regular and less complex spontaneous leg movements. Lower ApEn values were associated with higher lesion levels and later onset of independent walking, supporting ApEn as a sensitive marker of impaired neuromotor control.
	Surrogate Data Analysis	- Surrogate datasets generated using Chaos Data Analyzer software - LyE computed for original and surrogate signals - Determinism inferred when original LyE values differed significantly from surrogate values	Hip sagittal plane angle time series	Surrogate analysis confirmed that the hip angle time series were deterministic rather than random, validating the application of nonlinear analysis methods such as ApEn.
**Dusing et al. (2013, 2014, 2016) [62,63,64].**	ApEn	- m = 2; - Delay (τ) = 1 sample - Tolerance (r) = 0.2 × SD of the signal - Time series length: 500 samples (100 s)	CoP displacement time series (cranio–caudal, medio–lateral, and resultant)	2013: Postural control complexity was highest early in development and decreased as infants acquired midline head control and early reaching behaviors, particularly in the cranio–caudal direction. Changes in complexity occurred even when the magnitude of postural variability remained unchanged, supporting the role of temporal structure in the emergence of early motor behaviors. During early development. 2014: Infants born preterm exhibited significantly lower postural complexity, particularly in the medio–lateral direction, during the development of midline head control and reaching compared with infants born full term. Differences in complexity were evident before delays were detectable using standard clinical assessments, indicating that nonlinear measures captured subtle alterations in postural control strategies associated with prematurity. 2016: Infants born preterm demonstrated delayed adaptation of postural variability to task demands, particularly in the caudal–cephalic direction, while postural complexity remained unchanged across conditions and age. These findings indicate reduced adaptive postural control during early infancy, despite preservation of non-repetitive postural strategies.
**Gima et al. (2013) [61].**	FNN	- Optimal embedding dimension estimated using the FNN method - Delay (τ) = 250 ms (first zero of the autocorrelation function)	3D linear acceleration time series (x, y, z axes) of the right upper extremity	Infants with brain injuries exhibited significantly higher OED values at 3 months compared with healthy full-term infants, indicating increased dimensionality and altered organization of spontaneous upper extremity movements.
	Maximal Lyapunov Exponent (LyE)	- Kantz algorithm - m = 5; - Delay (τ) = 250 ms - Noise reduction applied prior to estimation	3D linear acceleration time series (x, y, z axes) of the right upper extremity	Positive LyE values were observed in both groups, confirming chaotic dynamics of spontaneous movements. However, infants with brain injuries showed uncorrelated developmental changes between OED and MLE across time, suggesting impaired self-organization and reduced coordination between motor system components.
**Aßmann et al. (2019) [65].**	Symbolic Dynamics	- Input signal: radial distance of hands and feet relative to trunk reference - Binary symbolic coding: 0 = proximal; 1 = distal. - Four limbs combined to generate 16 symbolic configurations - Configurations grouped into five configuration classes (I–V) based on spatial organization - Symbolic sequences analyzed continuously over time	3D kinematic trajectories of hands and feet	Symbolic dynamics revealed a small subset of configurations with high occurrence and recurrence, indicating the presence of preferred spatial organizations in spontaneous neonatal movements.
	Symbolic Recurrence Analysis	- Recurrence defined as repeated occurrence of identical symbolic configurations - Sliding window analysis applied to capture time-dependent changes in recurrence patterns - Recurrence quantified within and across configuration classes	Symbolic sequences derived from radial distance time series of hands and feet	Recurrence analysis demonstrated intermittent stabilization around specific configuration classes, alternating with exploratory phases, reflecting non-stationary and hierarchical organization of spontaneous motor behavior.
**Marchi et al. (2020) [23].**	Hjorth Parameters	- Input signals: velocity time series of hands and feet - Window length: 1 s, 50% overlap - First and last windows discarded to avoid edge effects - Hjorth parameters computed per window and averaged across windows, spatial components, and body side - Separate computation for upper and lower limbs	3D kinematic trajectories: velocity time series of hands and feet	Upper-limb Mobility decreased and Complexity increased with post-term age, indicating progressive stabilization and enrichment of movement patterns during fidgety age. Lower-limb Hjorth parameters showed no significant age associations.
**Wang et al. (2022) [66].**	Sample Entropy (SampEn)	- Embedding dimension (m): 3 - Tolerance (r): 0.1 × SD of the signal - Time series length: 100 s of CoP data - Algorithm: Richman & Moorman	Resultant CoP time series and derived velocity and acceleration signals	SampEn resultant CoP was significantly correlated with adjusted age, decreasing with increasing age, indicating reduced movement complexity as infants matured within the fidgety period. SampEn of CoP velocity and acceleration were not significantly associated with age.
**Shin et al. (2022) [67].**	SampEn	- m: not explicitly stated (consistent with standard SampEn implementations) - r: proportional to the standard deviation of the signal (exact value not specified) - SampEn computed separately for: ✓ Joint angle time series ✓ Joint angular velocity time series - Computation performed using R (pracma package)	Time series of joint angles and joint angular velocities extracted from 2D video-based pose estimation during spontaneous movements	Sample entropy values of joint angles and joint angular velocities were significantly lower in infants with HINE < 60 compared with those with HINE ≥ 60 and showed positive correlations with HINE scores across most upper- and lower-limb joints. These findings indicate reduced movement complexity in infants with poorer early neurological outcomes.
**Deng et al. (2023) [45].**	SampEn	- *m* = 2 - *r* = 0.2 × SD, continuous data length ≈ 10^4^ samples (per leg per day, 20 Hz, 8 h active data)	Lower-limb kinematic signals (ankle acceleration and angular velocity) recorded via IMUs during spontaneous movements	Infants in the AR group exhibited significantly lower SampEn values than the TD group, indicating reduced movement complexity and variability.
	Symbolic Dynamics (Barcoding States)	- Number of symbolic states: 16 - State probabilities calculated for each infant - SampEn applied to symbolic state sequences	Discrete symbolic representations of lower-limb acceleration and angular velocity time series	The AR group showed reduced diversity of symbolic states and lower entropy of state sequences, reflecting less adaptive and less complex spontaneous movement patterns than typically developing infants.
**Oh et al. (2024) [68].**	Fuzzy Entropy (FuzzyEn) Sample entropy–related rationale (comparative) Linear descriptive measures (supportive)	Embedding dimension (m) = 2; tolerance (r) = 0.2; applied to concatenated peak acceleration time series FuzzyEn chosen over SampEn to allow graded similarity (fuzzy membership function)	Peak acceleration per leg movement (m/s^2^), derived from wearable sensor data Lower-limb kinematic time series Average leg movement rate (mov/h); peak acceleration (m/s^2^)	Higher fuzzy entropy values were significantly associated with higher BSID-III motor composite scores at 12 months (median quantile regression), indicating greater movement complexity in infants with better motor outcomes Entropy of movement intensity (not movement quantity) emerged as the most sensitive predictor of motor development Neither movement rate nor peak acceleration alone predicted motor, language, or cognitive outcomes
**Park et al. (2024) [69].**	Sample Entropy (SampEn)	- *m* = 2 - *r* = 0.2 × SD; time series length ≈ 3000–7000 samples (24 Hz, 3–5 min) - SampEn computed separately for: ✓ Joint angle time series ✓ Joint angular velocity time series	Time series of joint angles and joint angular velocities derived from 2D video-based pose estimation during spontaneous infant movements	Infants with motor developmental delay exhibited significantly lower SampEn values across most upper- and lower-limb joints compared with infants without delay. SampEn measures showed positive correlations with motor composite scores of the BSID-III at 9 months corrected age, indicating reduced movement complexity in infants with poorer motor outcomes.

## References

[1] Marcroft C., Khan A., Embleton N.D., Trenell M., Plötz T. (2015). Movement Recognition Technology as a Method of Assessing Spontaneous General Movements in High Risk Infants. Front. Neurol..

[2] Miyagishima S., Asaka T., Kamatsuka K., Kozuka N., Kobayashi M., Igarashi L., Hori T., Tsutsumi H. (2018). Spontaneous Movements of Preterm Infants Is Associated with Outcome of Gross Motor Development. Brain Dev..

[3] Tacchino C., Impagliazzo M., Maggi E., Bertamino M., Blanchi I., Campone F., Durand P., Fato M., Giannoni P., Iandolo R. (2021). Spontaneous Movements in the Newborns: A Tool of Quantitative Video Analysis of Preterm Babies. Comput. Methods Programs Biomed..

[4] Hadders-Algra M. (2018). Early Human Motor Development: From Variation to the Ability to Vary and Adapt. Neurosci. Biobehav. Rev..

[5] Burger M., Louw Q.A. (2009). The Predictive Validity of General Movements—A Systematic Review. Eur. J. Paediatr. Neurol..

[6] Einspieler C., Bos A.F., Libertus M.E., Marschik P.B. (2016). The General Movement Assessment Helps Us to Identify Preterm Infants at Risk for Cognitive Dysfunction. Front. Psychol..

[7] Miyagishima S., Asaka T., Kamatsuka K., Kozuka N., Kobayashi M., Igarashi R., Hori T., Yoto Y., Tsutsumi H. (2016). Characteristics of Antigravity Spontaneous Movements in Preterm Infants up to 3 Months of Corrected Age. Infant Behav. Dev..

[8] Pires C.D.S., Marba S.T.M., Caldas J.P.D.S., Stopiglia M.D.C.S. (2020). Predictive Value of the General Movements Assessment in Preterm Infants: A Meta-Analysis. Rev. Paul. Pediatr..

[9] Einspieler C., Prechtl H.F.R., Ferrari F., Cioni G., Bos A.F. (1997). The Qualitative Assessment of General Movements in Preterm, Term and Young Infants–Review of the Methodology. Early Hum. Dev..

[10] Einspieler C., Peharz R., Marschik P.B. (2016). Fidgety Movements–Tiny in Appearance, but Huge in Impact. J. Pediatr..

[11] Robinson H., Hart D., Vollmer B. (2021). Predictive Validity of a Qualitative and Quantitative Prechtl’s General Movements Assessment at Term Age: Comparison between Preterm Infants and Term Infants with HIE. Early Hum. Dev..

[12] Waldmeier S., Grunt S., Delgado-Eckert E., Latzin P., Steinlin M., Fuhrer K., Frey U. (2013). Correlation Properties of Spontaneous Motor Activity in Healthy Infants: A New Computer-Assisted Method to Evaluate Neurological Maturation. Exp. Brain Res..

[13] Einspieler C., Marschik P.B., Pansy J., Scheuchenegger A., Krieber M., Yang H., Kornacka M.K., Rowinska E., Soloveichick M., Bos A.F. (2016). The General Movement Optimality Score: A Detailed Assessment of General Movements during Preterm and Term Age. Dev. Med. Child Neurol..

[14] Hadders-Algra M. (2004). General Movements: A Window for Early Identification of Children at High Risk for Developmental Disorders. J. Pediatr..

[15] Craciunoiu O., Holsti L. (2017). A Systematic Review of the Predictive Validity of Neurobehavioral Assessments During the Preterm Period. Phys. Occup. Ther. Pediatr..

[16] Kepenek-Varol B., Çalışkan M., İnce Z., Tatlı B., Eraslan E., Çoban A. (2016). The Comparison of General Movements Assessment and Neurological Examination during Early Infancy. Turk. J. Pediatr..

[17] Peinado Gorlat P., Gómez De Valcárcel Sabater M., Gorlat Sánchez B. (2020). Valoración de movimientos generales como herramienta pronóstica de parálisis cerebral infantil en prematuros: Revisión sistemática. Rev. Neurol..

[18] Rosendo N., Vericat A. (2023). Assessment of General Movements in Preterm Infants as a Predictor of Cerebral Palsy. Arch. Argent. Pediatr..

[19] Wu Y.-C., Straathof E.J.M., Heineman K.R., Hadders-Algra M. (2020). Typical General Movements at 2 to 4 Months: Movement Complexity, Fidgety Movements, and Their Associations with Risk Factors and SINDA Scores. Early Hum. Dev..

[20] Blankenship A.G., Feller M.B. (2010). Mechanisms Underlying Spontaneous Patterned Activity in Developing Neural Circuits. Nat. Rev. Neurosci..

[21] Dusing S.C., Harbourne R.T. (2010). Variability in Postural Control During Infancy: Implications for Development, Assessment, and Intervention. Phys. Ther..

[22] Haddersalgra M. (2007). Putative Neural Substrate of Normal and Abnormal General Movements. Neurosci. Biobehav. Rev..

[23] Marchi V., Belmonti V., Cecchi F., Coluccini M., Ghirri P., Grassi A., Sabatini A.M., Guzzetta A. (2020). Movement Analysis in Early Infancy: Towards a Motion Biomarker of Age. Early Hum. Dev..

[24] Ferrari F., Plessi C., Lucaccioni L., Bertoncelli N., Bedetti L., Ori L., Berardi A., Della Casa E., Iughetti L., D’Amico R. (2019). Motor and Postural Patterns Concomitant with General Movements Are Associated with Cerebral Palsy at Term and Fidgety Age in Preterm Infants. JCM.

[25] Hadders-Algra M. (2013). Typical and Atypical Development of Reaching and Postural Control in Infancy. Dev. Med. Child Neurol..

[26] Hadders-Algra M., Tacke U., Pietz J., Rupp A., Philippi H. (2024). Predictive Value of the General Movements Assessment and Standardized Infant NeuroDevelopmental Assessment in Infants at High Risk of Neurodevelopmental Disorders. Dev. Med. Child Neurol..

[27] Silva N., Zhang D., Kulvicius T., Gail A., Barreiros C., Lindstaedt S., Kraft M., Bölte S., Poustka L., Nielsen-Saines K. (2021). The Future of General Movement Assessment: The Role of Computer Vision and Machine Learning—A Scoping Review. Res. Dev. Disabil..

[28] Adde L., Rygg M., Lossius K., Øberg G.K., Støen R. (2007). General Movement Assessment: Predicting Cerebral Palsy in Clinical Practise. Early Hum. Dev..

[29] Adde L., Helbostad J.L., Jensenius A.R., Taraldsen G., Støen R. (2009). Using Computer-Based Video Analysis in the Study of Fidgety Movements. Early Hum. Dev..

[30] Adde L., Thomas N., John H.B., Oommen S., Vågen R.T., Fjørtoft T., Jensenius A.R., Støen R. (2016). Early Motor Repertoire in Very Low Birth Weight Infants in India Is Associated with Motor Development at One Year. Eur. J. Paediatr. Neurol..

[31] Adde L., Yang H., Sæther R., Jensenius A.R., Ihlen E., Cao J., Støen R. (2018). Characteristics of General Movements in Preterm Infants Assessed by Computer-Based Video Analysis. Physiother. Theory Pract..

[32] Disselhorst-Klug C., Heinze F., Breitbach-Faller N., Schmitz-Rode T., Rau G. (2012). Introduction of a Method for Quantitative Evaluation of Spontaneous Motor Activity Development with Age in Infants. Exp. Brain Res..

[33] Fetters L., Sapir I., Chen Y., Kubo M., Tronick E. (2010). Spontaneous Kicking in Full-term and Preterm Infants with and without White Matter Disorder. Dev. Psychobiol..

[34] Grant-Beuttler M., Heriza C.B., Palisano R.J., Wagner B.R., Miller D.P., Karduna A. (2016). Ankle Movements During Supine Kicking in Infants Born Preterm. Pediatr. Phys. Ther..

[35] Halek J., Muckova A., Svoboda Z., Janura M., Marikova J., Horakova K., Kantor L., Nemcova N. (2015). Kinematic Analysis of Preterm Newborns’ Spontaneous Movements for Postural Activity Assessment.

[36] Heinze F., Hesels K., Breitbach-Faller N., Schmitz-Rode T., Disselhorst-Klug C. (2010). Movement Analysis by Accelerometry of Newborns and Infants for the Early Detection of Movement Disorders Due to Infantile Cerebral Palsy. Med. Biol. Eng. Comput..

[37] Jan Y.-K., Lin C.-F., Liao F., Singh N.B. (2023). Editorial: Nonlinear Dynamics and Complex Patterns in the Human Musculoskeletal System and Movement. Front. Bioeng. Biotechnol..

[38] Lee M., Ranganathan R., Newell K.M. (2011). Changes in Object-oriented Arm Movements That Precede the Transition to Goal-directed Reaching in Infancy. Dev. Psychobiol..

[39] Støen R., Songstad N.T., Silberg I.E., Fjørtoft T., Jensenius A.R., Adde L., On behalf of the CIMA Norway Study Group (2017). Computer-Based Video Analysis Identifies Infants with Absence of Fidgety Movements. Pediatr. Res..

[40] Harbourne R.T., Stergiou N. (2009). Movement Variability and the Use of Nonlinear Tools: Principles to Guide Physical Therapist Practice. Phys. Ther..

[41] Caballero C., Barbado D., Moreno F. (2014). Non-Linear Tools and Methodological Concerns Measuring Human Movement Variability: An Overview. Eur. J. Hum. Mov..

[42] Page M.J., McKenzie J.E., Bossuyt P.M., Boutron I., Hoffmann T.C., Mulrow C.D., Shamseer L., Tetzlaff J.M., Akl E.A., Brennan S.E. (2021). The PRISMA 2020 Statement: An Updated Guideline for Reporting Systematic Reviews. J. Clin. Epidemiol..

[43] Aßmann B., Thiel M., Romano M.C., Niemitz C. (2006). Recurrence Plot Analyses Suggest a Novel Reference System Involved in Newborn Spontaneous Movements. Behav. Res. Methods.

[44] Aßmann B., Romano M.C., Thiel M., Niemitz C. (2007). Hierarchical Organization of a Reference System in Newborn Spontaneous Movements. Infant Behav. Dev..

[45] Deng W., Marmelat V., Vanderbilt D.L., Gennaro F., Smith B.A. (2023). Barcoding, Linear and Nonlinear Analysis of Full-day Leg Movements in Infants with Typical Development and Infants at Risk of Developmental Disabilities: Cross-sectional Study. Infancy.

[46] Hesse N., Bodensteiner C., Arens M., Hofmann U.G., Weinberger R., Sebastian Schroeder A., Leal-Taixé L., Roth S. (2019). Computer Vision for Medical Infant Motion Analysis: State of the Art and RGB-D Data Set. Computer Vision—ECCV 2018 Workshops.

[47] Kędziorek J., Błażkiewicz M. (2020). Nonlinear Measures to Evaluate Upright Postural Stability: A Systematic Review. Entropy.

[48] Smith B., Vanderbilt D., Applequist B., Kyvelidou A. (2017). Sample Entropy Identifies Differences in Spontaneous Leg Movement Behavior between Infants with Typical Development and Infants at Risk of Developmental Delay. Technologies.

[49] Winter L., Taylor P., Bellenger C., Grimshaw P., Crowther R.G. (2023). The Application of the Lyapunov Exponent to Analyse Human Performance: A Systematic Review. J. Sports Sci..

[50] Zuk L. (2011). Fetal and Infant Spontaneous General Movements as Predictors of Developmental Disabilities. Dev. Disabil. Res. Rev..

[51] Freitas M., Pinho F., Pinho L., Silva S., Figueira V., Vilas-Boas J.P., Silva A. (2024). Biomechanical Assessment Methods Used in Chronic Stroke: A Scoping Review of Non-Linear Approaches. Sensors.

[52] Kieszczyńska K., Doroniewicz I., Ledwoń D., Bugdol M., Affanasowicz A., Latos D., Matyja M., Myśliwiec A. (2024). Reproducibility Evaluation of Kinesiological Parameters Spontaneous Movements of Infants in Computer Analysis. Acta Bioeng. Biomech..

[53] Tsuji T., Nakashima S., Hayashi H., Soh Z., Furui A., Shibanoki T., Shima K., Shimatani K. (2020). Markerless Measurement and Evaluation of General Movements in Infants. Sci. Rep..

[54] Tricco A.C., Lillie E., Zarin W., O’Brien K.K., Colquhoun H., Levac D., Moher D., Peters M.D.J., Horsley T., Weeks L. (2018). PRISMA Extension for Scoping Reviews (PRISMA-ScR): Checklist and Explanation. Ann. Intern. Med..

[55] Peters M.D.J., Marnie C., Tricco A.C., Pollock D., Munn Z., Alexander L., McInerney P., Godfrey C.M., Khalil H. (2020). Updated Methodological Guidance for the Conduct of Scoping Reviews. JBI Evid. Synth..

[56] Ohgi S., Morita S., Loo K.K., Mizuike C. (2007). A Dynamical Systems Analysis of Spontaneous Movements in Newborn Infants. J. Mot. Behav..

[57] Ohgi S., Morita S., Loo K.K., Mizuike C. (2008). Time Series Analysis of Spontaneous Upper-Extremity Movements of Premature Infants With Brain Injuries. Phys. Ther..

[58] Dusing S.C., Kyvelidou A., Mercer V.S., Stergiou N. (2009). Infants Born Preterm Exhibit Different Patterns of Center-of-Pressure Movement Than Infants Born at Full Term. Phys. Ther..

[59] Gima H., Ohgi S., Morita S., Karasuno H., Fujiwara T., Abe K. (2011). A Dynamical System Analysis of the Development of Spontaneous Lower Extremity Movements in Newborn and Young Infants. J. Physiol. Anthropol..

[60] Smith B.A., Teulier C., Sansom J., Stergiou N., Ulrich B.D. (2011). Approximate Entropy Values Demonstrate Impaired Neuromotor Control of Spontaneous Leg Activity in Infants with Myelomeningocele. Pediatr. Phys. Ther..

[61] Gima H., Ohgi S., Morita S., Karasuno H., Fujiwara T., Abe K. (2013). A Comparison of the Developmental Characteristics of Spontaneous Upper Extremity Movements between Healthy Full-Term Infants and Premature Infants with Brain Injuries. J. Appl. Biometrol..

[62] Dusing S.C., Thacker L.R., Stergiou N., Galloway J.C. (2013). Early Complexity Supports Development of Motor Behaviors in the First Months of Life. Dev. Psychobiol..

[63] Dusing S.C., Izzo T.A., Thacker L.R., Galloway J.C. (2014). Postural Complexity Differs between Infant Born Full Term and Preterm during the Development of Early Behaviors. Early Hum. Dev..

[64] Dusing S.C., Thacker L.R., Galloway J.C. (2016). Infant Born Preterm Have Delayed Development of Adaptive Postural Control in the First 5 Months of Life. Infant Behav. Dev..

[65] Assmann B., Kaese T., Neumeister A., Disselhorst-Klug C. (2019). Self-Organization in Spontaneous Movements of Neonates Generates Self-Specifying Sensory Experiences. arXiv.

[66] Wang J., Siddicky S.F., Johnson T., Kapil N., Majmudar B., Mannen E.M. (2021). Supine Lying Center of Pressure Movement Characteristics as a Predictor of Normal Developmental Stages in Early Infancy. THC.

[67] Shin H.I., Shin H.-I., Bang M.S., Kim D.-K., Shin S.H., Kim E.-K., Kim Y.-J., Lee E.S., Park S.G., Ji H.M. (2022). Deep Learning-Based Quantitative Analyses of Spontaneous Movements and Their Association with Early Neurological Development in Preterm Infants. Sci. Rep..

[68] Oh J., Ordoñez E.L.T., Velasquez E., Mejía M., Del Pilar Grazioso M., Rohloff P., Smith B.A. (2024). Associating Neuromotor Outcomes at 12 Months with Wearable Sensor Measures Collected during Early Infancy in Rural Guatemala. Gait Posture.

[69] Park M.W., Shin H.-I., Bang M.S., Kim D.-K., Shin S.H., Kim E.-K., Lee E.S., Shin H.I., Lee W.H. (2024). Reduction in Limb-Movement Complexity at Term-Equivalent Age Is Associated with Motor Developmental Delay in Very-Preterm or Very-Low-Birth-Weight Infants. Sci. Rep..

[70] Saha S., Pagnozzi A., Bourgeat P., George J.M., Bradford D., Colditz P.B., Boyd R.N., Rose S.E., Fripp J., Pannek K. (2020). Predicting Motor Outcome in Preterm Infants from Very Early Brain Diffusion MRI Using a Deep Learning Convolutional Neural Network (CNN) Model. NeuroImage.

[71] Bortagarai F.M., Moraes A.B.D., Pichini F.D.S., Souza A.P.R.D. (2021). Risk Factors for Fine and Gross Motor Development in Preterm and Term Infants. CoDAS.

[72] Elgendy M.M., Puthuraya S., LoPiccolo C., Liu W., Aly H., Karnati S. (2022). Neonatal Stroke: Clinical Characteristics and Neurodevelopmental Outcomes. Pediatr. Neonatol..

[73] Hadders-Algra M. (2010). Variation and Variability: Key Words in Human Motor Development. Phys. Ther..

[74] Latash M.L. (2012). The Bliss (Not the Problem) of Motor Abundance (Not Redundancy). Exp. Brain Res..

[75] Tang Y., Triesch J., Deák G.O. (2023). Variability in Infant Social Responsiveness: Age and Situational Differences in Attention-Following. Dev. Cogn. Neurosci..

[76] Parks M.T., Wang Z., Siu K.-C. (2019). Current Low-Cost Video-Based Motion Analysis Options for Clinical Rehabilitation: A Systematic Review. Phys. Ther..

[77] Gu B., Kim H.S., Kim H., Yoo J.-I. (2026). Advancements in Wearable Sensor Technologies for Health Monitoring in Terms of Clinical Applications, Rehabilitation, and Disease Risk Assessment: Systematic Review. JMIR mHealth uHealth.

[78] Patel S., Park H., Bonato P., Chan L., Rodgers M. (2012). A Review of Wearable Sensors and Systems with Application in Rehabilitation. J. Neuroeng. Rehabil..

[79] Farabolini G., Baldini N., Pagano A., Andrenelli E., Pepa L., Morone G., Ceravolo M.G., Capecci M. (2025). Continuous Movement Monitoring at Home Through Wearable Devices: A Systematic Review. Sensors.

[80] Stergiou N. (2016). Nonlinear Analysis for Human Movement Variability.

[81] Błażkiewicz M., Hadamus A., Borkowski R. (2023). Recurrence Quantification Analysis as a Form of Postural Control Assessment: A Systematic Review. Appl. Sci..

[82] Hirata Y., Amigó J.M. (2023). A Review of Symbolic Dynamics and Symbolic Reconstruction of Dynamical Systems. Chaos Interdiscip. J. Nonlinear Sci..

[83] Deffeyes J.E., Harbourne R.T., DeJong S.L., Kyvelidou A., Stuberg W.A., Stergiou N. (2009). Use of Information Entropy Measures of Sitting Postural Sway to Quantify Developmental Delay in Infants. J. Neuroeng. Rehabil..

[84] Cofré Lizama L.E., He X., Kalincik T., Galea M.P., Panisset M.G. (2024). Sample Entropy Improves Assessment of Postural Control in Early-Stage Multiple Sclerosis. Sensors.

[85] Chen W., Zhuang J., Yu W., Wang Z. (2009). Measuring Complexity Using FuzzyEn, ApEn, and SampEn. Med. Eng. Phys..

[86] Platakis D., Manis G. (2025). Review of Recent (2015–2024) Popular Entropy Definitions Applied to Physiological Signals. Entropy.

[87] Azami H., Li P., Arnold S.E., Escudero J., Humeau-Heurtier A. (2019). Fuzzy Entropy Metrics for the Analysis of Biomedical Signals: Assessment and Comparison. IEEE Access.

